# Supplementary material for: Accelerated, Dose escalated, Sequential Chemoradiotherapy in Non-small-cell lung cancer (ADSCaN): a protocol for a randomised phase II study
Source: BMJ Open. 2019 Jan 29;9(1):e019903. doi: 10.1136/bmjopen-2017-019903 (PMC6352760; doi:10.1136/bmjopen-2017-019903)
Supplement: Supplementary data [file bmjopen-2017-019903supp001.pdf]

(Form to be on hospital headed paper)

INFORMATION SHEET FOR PATIENTS/ VOLUNTEERS IN CLINICAL RESEARCH PROJECT

Title of Project:
ADSCaN - A Randomised Phase II trial of **A**ccelerated, **D**ose escalated, **S**equential **C**hemo-r**a**diotherapy in **N**on-small cell lung cancer

Invitation Paragraph

We would like to invite you to take part in a research study (also called a clinical trial) because your doctors are considering a course of radiotherapy for your lung cancer. Before you decide whether or not to take part, it is important for you to understand why the research is being done and what it will involve for you. Please take time to read the following information carefully and discuss it with friends, family or your General Practitioner (GP) if you wish. Ask us if there is anything that is not clear or if you would like more information. Take time to decide whether or not you wish to take part.

Summary

When we refer to 'your doctor' in this information sheet we are referring to the consultant oncologist responsible for your care relating to your lung cancer in hospital.

Your doctor will have explained that the combination of chemotherapy followed by radiotherapy is the best treatment for your cancer.

You may already have started chemotherapy. Radiotherapy treats the cancer by using high-energy x-rays to destroy cancer cells. This treatment is given in hospital radiotherapy departments in short sessions that only take a few minutes, each session is known as a fraction. The fractions add up to a full course of treatment.

It is thought that giving a higher dose of radiotherapy during the course of treatment will destroy more of the cancer cells, but this has to be done with no more measurable damage to the nearby healthy cells.

Part 1 tells you the purpose of this trial and what will happen to you if you take part.

Part 2 gives you more detailed information about the conduct of the trial.

PART 1

What is the purpose of this trial?

You have been diagnosed with a certain form of lung cancer, called non-small cell lung cancer (NSCLC). Patients with locally advanced lung cancer are often treated with chemotherapy and high dose radiotherapy when surgery is not an option. This trial is looking at different courses of radiotherapy treatment for patients with non-small cell lung cancer. In order to try to improve the result of treatment a number of UK trial

groups have been developing higher dose radiotherapy courses and testing their safety. Four courses that increase the radiotherapy dose delivered to the tumour have been developed and trials have now shown us the right dose for these courses. We now wish to test these different radiotherapy courses against each other to see which is most promising and compare them to the current UK standard course of treatment.

In some of these new courses of treatment, the fractions are given once a day and in others there are two or three fractions per day. This new study will try to identify which course of treatment is the most promising and should be tested further in a future trial; the ultimate aim is to increase the cure rate for patients with inoperable lung cancer. Your hospital is able to offer one or more of these new treatment courses.

Why have I been invited?

You have been invited to take part in this trial because you have non-small cell lung cancer which can be treated with radiotherapy after a course of chemotherapy.

Do I have to take part?

Taking part in the trial is entirely voluntary. It is up to you to decide whether or not to take part. If you do decide to take part, you will be given this information sheet to keep and asked to sign a consent form.

If you decide to take part you are still free to withdraw at any time and without giving a reason. If you do not wish to participate this will not affect the standard of your treatment and you will be offered whatever treatment your consultant thinks is best for you.

How are treatment options decided?

When a treatment is discovered we don't always know which way of treating patients is best. To find out, we need to make comparisons between the different treatments. To do this we put patients randomly into groups and give each group a different treatment; the results are compared to see if one treatment is better than the others.

To try to make sure the groups are the same to start with, each patient is put into a group by chance. This process is called randomisation and, if you agree to take part, you will be put into a group by chance when neither you, nor your doctor can pick the treatment group. You will receive one of the following ticked radiotherapy treatment course *(the treatment courses available at this hospital are ticked)*:

<< TO BE POPULATED AS PER THE ARMS SITE IS PARTICIPATING IN >>

- ☑Standard Radiotherapy
- ☐CHART-ED
- ☐IDEAL
- ☐I-START
- ☐ISOTOXIC IMRT

In this trial we are aiming to randomise 120 patients to standard radiotherapy and 60 patients to each of the four new courses of radiotherapy: CHART-ED, IDEAL, I-START, ISOTOXIC IMRT.

What will happen to me if I take part?

Standard radiotherapy is given over 4 weeks with 20 fractions given on Monday to Friday. If you agree to take part in the study, the length of the course of radiotherapy will vary so your course may be slightly shorter or longer than the standard lasting between 17 days and 5 weeks in total.

Before starting radiotherapy treatment, we will check your general health and weight, which includes taking a history of your previous health and current medications and carry out a physical examination. You will have all of the normal tests that are performed before radiotherapy; these include a CT scan of your chest and abdomen, chest x-ray, blood tests, breathing tests (Pulmonary Function Tests) and a heart trace (ECG). You will also have a CT or MRI scan of your head that may be an additional test done for the trial. These tests are done to make sure you are suitable for the trial.

You will then attend the Radiotherapy Department for a planning-CT scan. The doctors, radiographers and physicists will use this CT scan to plan your treatment. Once the radiotherapy has been planned, you will attend the Radiotherapy Department for a "planning" session. If this is satisfactory, your radiotherapy treatment will start a few days later.

If any results of the tests carried out mean you cannot participate in the trial, you will be unable to enter the trial and your treating doctor will ensure that you will receive the standard treatment that is best for you.

At some visits to hospital your doctor or nurse will ask you some extra questions about how you are feeling and if you have noticed any side effects from your radiotherapy. After any radiotherapy treatment, you will continue to be seen by your doctor to check for any side effects and see how well your treatment has worked. You will see your doctor in clinic as you would normally, but you may be asked some additional questions at these visits.

You will receive a course of radiotherapy treatment outlined in the table:

**Course of Radiotherapy**	**Description**	**Sessions per day**	**Number of Weeks**
**Standard Radiotherapy**	One radiotherapy session per day on weekdays over 4 weeks.	1	4
**CHART-ED**	Three radiotherapy sessions per day over 12 days (including weekend) without a gap. This is followed after a weekend gap of 2 days by two radiotherapy sessions per day over 3 days.<br><br>Treatment will last 17 days in total. It will start on a Monday and you will stay in hospital whilst you receive treatment. You will have a break and be allowed to go home for the second weekend (get home on the Friday afternoon and return on the Monday morning).	3	2.5
**IDEAL**	One-to-two radiotherapy sessions per day on weekdays over 5 weeks.	1	5
**I-START**	One radiotherapy session per day on weekdays over 4 weeks.	1	4
**ISOTOXIC IMRT**	Two radiotherapy sessions per day on weekdays over 4 weeks.<br><br>You will be treated with indivdualised (isotoxic and targeted (IMRT) doses of radiotherapy. The radiotherapy session will take around 15 minutes each time and there will be a break of at least 6 hours between the two sessions given each day.<br><br>If you do not live close enough to the hospital to return home during the break between radiotherapy sessions, you may be able to stay in hospital.	2	4

You will have blood tests whilst you are receiving the radiotherapy, which would happen as part of your standard care. 1-2 teaspoons or 5-10ml of blood will be needed.

You will have your breathing tests repeated at three months and one year after you have finished your radiotherapy.

You will continue to have chest x-rays and CT scans regularly to make sure your lung cancer has not come back. The chest x-rays would all be done as part of your standard care. You will have a CT scan at 3, 6, 12 18, 24 and 36 months after radiotherapy. You would have the CT scans at 3 and 12 months after radiotherapy as part of your standard care, however in this trial you will have the additional scans so that we can check your response to radiotherapy more closely.

In addition, we will ask you to fill out questionnaires called “Quality of Life” questionnaires before the beginning of the trial, during radiotherapy treatment and at every clinic visit after treatment. These questionnaires ask you about how you feel and take about 10 minutes to complete. Some of these questions are personal and you can refuse to answer them if you wish. The information you provide is for research purposes and will remain strictly private and confidential.

What is the standard treatment?
The standard radiotherapy treatment most commonly used in the UK is 20 fractions given over 28 days. This is a lower dose than those of the experimental arms being tested in this trial.

What are the possible benefits of taking part?
The information we get from this trial may help us to decide if some radiotherapy treatments are better than others are and may help us to treat future patients with the same disease better.

What are the possible disadvantages and risks of taking part?
There are side effects associated with the radiotherapy, which are detailed below. If you are randomised to receive experimental radiotherapy these doses are slightly higher than the standard dose, therefore side effects may be more likely to occur or may be more severe. You can stop the trial treatment at any time if you or your doctor feels that the side effects are a problem for you.

Information on side effects and the treatment of these side effects will be closely monitored by doctors involved in the trial. In addition, the trial will be carefully monitored by an independent group of specialists who will regularly advise all the investigators about the safety of the trial.

As part of this trial, you will be exposed to radiation from computed tomography (CT) scans and from your radiotherapy treatment. Your radiotherapy treatment uses strong (high energy) X-rays to treat your cancer, whereas CT scans use lower energy X-rays to form pictures of your body, which provide detailed information about your cancer. The dose of radiation you receive from a CT scan is much less than the dose of radiation you will receive from your radiotherapy.

The risk of radiation exposure associated with chest x-ray is extremely small and relates to a lifetime risk of induced cancer of 0.0005%, which is 5 in one million.

The total radiation dose due to all forms of imaging examinations carried out in the course of the study corresponds to an estimated lifetime risk of induced cancer of 0.3 – 1.2%. Of this total imaging dose, around two-thirds comes from examinations that would be carried out in the course of normal treatment anyway. The remaining non-routine dose corresponds to a lifetime induced cancer risk of 0.01 – 0.67%.

In a previous lung radiotherapy study that delivered escalated doses, cancers most probably induced directly by the radiation treatment were seen in 2 of 112 patients.

There are no radiation risks associated with MRI. However, some people are allergic to the contrast fluid that is injected as part of the CT or MRI scan procedure. Sometimes contrast fluid can cause damage to the kidneys. Both of these are rare reactions. If you have previously had a bad reaction to contrast fluid, please let your doctor know.

You may be given a higher dose of radiotherapy so the side effects may be more than if you had the standard dose. This is monitored extremely carefully to ensure the amount received by normal tissues is within safe limits and is also safe for you. You will need to tell your doctor or nurse about any side effects you experience.

What are the side effects of radiotherapy?

Early side effects

These can occur during the four weeks of radiotherapy or soon after the treatment has finished. They almost always settle within 2 months of finishing radiotherapy.

Soreness when swallowing (Oesophagitis) - this can start towards the middle of treatment and may be at its worst a week or two after treatment has finished. It should then gradually settle down. There are medicines available that may help this.

Cough and shortness of breath - the radiotherapy may cause inflammation in the lung which can cause shortness of breath and cough. There are medicines which may help control these symptoms.

Skin reaction – skin over the treated area may become red as the treatment progresses.

Tiredness – you will probably feel more tired than usual. This will wear off gradually, but may last for a number of weeks after treatment finishes.

Late side effects

These can occur several months after the radiotherapy has finished. They are usually permanent.

Soreness and difficulty swallowing - Unusually, radiotherapy can cause scarring and narrowing of the oesophagus. This can be helped by medicine but may need further investigation and treatment

Lung scarring (Fibrosis) – The radiotherapy will damage some of the normal lung tissue around the cancer which will, after six to nine months, cause some scarring. This can cause breathlessness, which in a minority of cases can be severe.

Skin colour change – Sometimes the skin over the treated area is left darker or lighter than it was before the radiotherapy. You will receive information about skin care when you start your treatment.

Effect of treatment on fertility and contraception

Radiotherapy can cause damage to the ovaries or testes. This may cause permanent infertility (inability to have a child) but the risk is very small when the lungs are being treated.

If it is possible you may conceive (i.e. you are of childbearing age), you will be asked to have a pregnancy test before starting radiotherapy treatment.

It is still possible for women of childbearing age to become pregnant, and men to father a child during treatment. Therefore, you or your partner should use effective barrier methods of contraception during treatment (e.g. condoms), and for six months after treatment, because of the risk to the unborn child. You would be advised to do this even if you were not taking part in this trial. In the event that you or your partner becomes pregnant while you are taking part in the trial you should consult your doctor immediately and inform your research nurse. Your research nurse will then report the pregnancy to the Cancer Research UK Clinical Trials Unit Glasgow, who are running the trial. Should you or your partner become pregnant during the trial you will be provided with additional information about this process.

What are the risks from radiation?

As part of this trial, you will be exposed to radiation from computed tomography (CT) scans and from your radiotherapy treatment. Your radiotherapy treatment uses strong (high energy) X-rays to treat your cancer. CT scans use lower energy X-rays to form pictures of your body which provide detailed information about your cancer. The dose of radiation you receive from a CT scan is much less than the dose of radiation you will receive from your radiotherapy.

As part of this trial, you will have more scans than with standard care. This additional radiation may slightly increase the risk of your developing cancer at a later date. However the risk is considered to be small when compared to the possible benefits of treatment.

In addition to these risks, some people are allergic to the contrast which is injected at the time of the CT scans. Sometimes contrast can cause damage to the kidneys. If you have previously had a reaction like this, please let your doctor know.

What happens when the research trial stops?

If the research trial stops and you require treatment you will receive the standard radiotherapy dose you would have been given if you had not agreed to take part in the trial.

If you have already received radiotherapy treatment you will continue to be followed up in the normal way.

What if there is a problem?
Any complaint about the way you have been dealt with during the trial or any possible harm you might suffer will be addressed. The detailed information concerning this is given in Part 2 of this information sheet. If you have any concerns or complaints you should contact your doctor in the first instance.

Will my taking part in the trial be kept confidential?
Yes. We will follow ethical and legal practices and all information about you will be handled in confidence. The details are included in Part 2.

This completes Part 1 of the information sheet.

If the information in Part 1 has interested you and you are considering taking part, please read the additional information in Part 2 before making any decision.

PART 2

What if I change my mind?
If you do decide to take part in this trial then you are still free to withdraw at any time without giving a reason. This will not affect your future care in any way but we would still like to know how you are, and with your permission, will request this information from your doctor. Please let your doctor know if you wish to withdraw and he/she will carry on your care in the normal way.
If you withdraw, you will be asked to clarify which part of the trial you are withdrawing from. You may withdraw from some parts or all parts. Depending on your response, we will know what information we can keep and whether we can still collect information on your progress.

What if new information becomes available?
Sometimes during the course of a research project, new information becomes available about the treatment that is being studied. If this happens, your doctor will tell you about it and discuss with you whether you want to continue in the trial. If you decide to withdraw, your doctor will make arrangements for your normal care to continue. If you decide to continue in the trial you will be asked to sign a new consent form.

Also, on receiving new information, your doctor might consider it to be in your best interest to withdraw you from the trial. He/she will explain the reasons and arrange for your usual care to continue.

What if something goes wrong?
If you have a concern about any aspect of this trial, you should ask to speak with the research doctor/nurse, who will do their best to answer your questions.

If taking part in this research trial harms you, there are no special compensation arrangements. If you are harmed due to someone's negligence, then you may have grounds for a legal action but you may have to pay your legal costs. Regardless of this, if you wish to complain or have any concerns about any aspect of the way you have been approached or treated during the course of this trial, the normal National Health Service complaints mechanism is available to you.

If you have private medical insurance, you may wish to check with your company before agreeing to take part in the trial to ensure that participation in the trial will not affect your insurance cover.

Additional Research – translational studies

We will be asking you whether we can collect some samples to allow us to carry out further research studies. These samples will include a sample of the original tumour taken at the time of your original biopsy, and will have been stored in the pathology department since then. We may also ask you for a blood test, which could be carried out at the same time as routine blood tests.

Samples from patients participating in clinical trials such as ADSCaN, are extremely valuable as accurate clinical information is collected on the same patients. This information, including whether the cancer responds to treatment or whether the disease comes back in the future, can be used together with the information discovered in the laboratory to help answer questions about lung cancer and its treatment.

These additional studies will not affect your treatment in any way. If you decide not to take part in the collection of samples for laboratory research, this will not affect your participation in the clinical part of ADSCaN, or your relationship with your doctor.

Any unused parts of the samples collected will be stored for as long as possible, in case further investigations are developed in the future, which may help us understand more about the response of your type of cancer to these therapies. The samples may also be used for other studies in the future relating to lung cancer, and may involve genetic tests to look at genetic influences on these cancer types. Any such studies will have to be approved by the ADSCaN Trial Management Group and external ethics committees. We cannot describe here all the potential tests which may be done on any samples stored and if you feel uncomfortable about this please do not participate.

Will my taking part in this trial be kept confidential?

You can be assured that any data collected during the course of this trial and any of the results published will not identify you personally. Your medical records will only be available to the research doctors, your hospital consultant, responsible individuals from the Cancer Research UK Clinical Trials Unit (Glasgow), trial sponsors and the regulatory authority.

With your permission, we will also inform your general practitioner of your participation in this trial.

With your permission, the Cancer Research UK Clinical Trials Unit (Glasgow) who co-ordinate the participation in the trial, will collect your name or initials, date of birth and NHS number or Community Health Index (CHI) number. This information will be stored securely on a password-protected database and will be kept strictly confidential, with access provided only to authorised personnel. This information will utilize NHS data for future research through Cancer Research UK, who are funding this trial.

Your consent for participation in this trial also includes your consent to allow the use of the data in your medical/clinical record to be used for the purposes of cancer research. Your consent also includes allowing this data to be linked to data coming from other sources such as cancer registries and medical clinical records. All data (personal, clinical, economic and data coming from research on biological material) collected on your behalf will be treated in compliance with the European and UK applicable laws to ensure your confidentiality is maintained.

What will happen to the results of the research trial?
The results of this trial will be presented at medical meetings and submitted to major cancer research journals for publication. You will not be identified in any way in any report or publication arising from the trial. Results will also be available through http://cancerbackup.org.uk

The results of this trial will also be presented outside the European Union. These areas may have fewer rules about data protection. However, you would never be identified individually during these presentations. Data sent to other groups in the UK and abroad will not include information that identifies you by name, and agreements will ensure the data is treated confidentially.

Who is organising and funding the research?
The research is being sponsored by NHS Greater Glasgow and Clyde and is funded by Cancer Research UK. The trial is being co-ordinated by the Cancer Research UK Clinical Trials Unit, Glasgow on behalf of the NHS Greater Glasgow and Clyde. The Chief Investigator is Dr Matthew Hatton, Consultant Clinical Oncologist at Weston Park Hospital, Sheffield.

Will I be paid any costs and reimbursements?
There will be no payment to you for entering this study, no reimbursement of expenses and no payment for undergoing investigations that are additional to your standard care.

Who has reviewed this trial?
This clinical trial has been reviewed by number of medical specialists during its development; the National Cancer Research Institute Lung Clinical Studies Group (NCRI Lung CSG) and the Clinical Trials Advisory and Awards Committee in the UK (CTAAC).

All research in the NHS is looked at by an independent group of people, called a Research Ethics Committee, to protect your interests. This trial has been reviewed and given a favourable opinion by the West of Scotland Research Ethics Committee.

Further information and contact details

If you consent to join this trial you will be given the telephone number of the hospital, which you can contact at any time if you feel unwell or have questions. We would like you to keep this information sheet and if you find it helpful please use it to discuss treatment with your family, friends or your General Practitioner. If you have any further questions or concerns regarding the trial please contact:

Doctor:

**Name**	**Insert local details**
**Telephone Number**	**Insert local details** (During office hours) or if out of office hours call **(Insert Local Out of Hours/Emergency Contact Details)**

Research Nurse:

**Name**	**Insert local details**
**Telephone Number**	**Insert local details**

If you would like independent advice or further information you may also find it useful to contact;

Cancerhelp UK (Cancer Research UK), who provide a wide range of information for people with cancer: Freephone: 0808 800 4040, and website: www.cancerhelp.org.uk

Macmillan Cancer Support, an independent patient advisory group: Freephone 0808 808 00 00; website www.macmillan.org.uk, Head Office Address: Macmillan Cancer Support, 89 Albert Embankment, London, SE1 7UQ.

Thank you for taking the time to read this information sheet and consider this trial. If you wish to take part you will be given a copy of this information sheet and a signed consent form to keep.

ADSCaN PARTICIPANT CONSENT FORM

Patient ID Number for this trial: Date:

Name of Principal Investigator:

Please initial box

1. I confirm that I have read and understand the information sheet dated (Version XXX Date XXX) for the above trial and have had the opportunity to ask questions.

2. I understand that my participation is voluntary and that I am free to withdraw at any time, without giving any reason, without my medical care or legal rights being affected.

3. I agree that relevant sections of any of my medical notes and data collected during the trial may be looked at by responsible individuals from the Cancer Research UK Clinical Trials Unit (Glasgow), the trial sponsors, the NHS organisation (hospital site) and the regulatory authorities where it is relevant to my taking part in research. I give permission for these individuals to have access to my records.

4. I consent to the storage of personal information (including electronic) for the purposes of this trial. I understand that any information that could identify me will be kept strictly confidential and that no personal information will be included in the trial report or other publication.

5. I agree to give permission for data collected relating to me to be used for "cancer research" purposes as described in this information sheet including allowing this data to be linked to data coming from other sources such as cancer registries and medical clinical records. I understand giving consent to the use of this data as described is optional and not mandatory for participating in this trial.

6. I give permission for my initials, date of birth and NHS (or Community Health Index (CHI)) number to be collected by the Cancer Research UK Clinical Trials Unit (Glasgow), where they will be stored in a secure location (including electronic). I understand giving consent to the use of this data as described is optional and not mandatory for participating in this trial.

7. I give permission for my GP to be informed of my inclusion in this trial.

8. I agree to give extra samples of blood and tissue for translational research purposes as described in the information sheet for this trial. I understand how the samples will be collected, that giving samples is voluntary and that I am free to withdraw my approval for use of the samples at any time without giving a reason and without my medical care or legal rights being affected **(optional)**. ☐

9. I agree that the blood and tissue samples and information collected about me will be stored on behalf of the ADSCaN Trial Management Group and may be used in future projects, as described in the information sheet. I understand that some of these projects may be carried out by researchers other than the ADSCaN Trial Management Group **(optional)**. ☐

10. I understand that I shall not benefit financially if future research leads to the development of new treatments or medical tests. ☐

11. I agree to the information detailed in the information sheet to be collected as part of the trial. ☐

12. I agree to take part in the ADSCaN trial. ☐

Name of Patient	Date	Signature
Name of person taking consent	Date	Signature

1 for patient; 1 for researcher; 1 to be kept with hospital notes
