# Supplementary material for: Accelerated, Dose escalated, Sequential Chemoradiotherapy in Non-small-cell lung cancer (ADSCaN): a protocol for a randomised phase II study
Source: BMJ Open. 2019 Jan 29;9(1):e019903. doi: 10.1136/bmjopen-2017-019903 (PMC6352760; doi:10.1136/bmjopen-2017-019903)
Supplement: Author's manuscript [file bmjopen-2017-019903.draft_revisions.pdf]

BMJ Open is committed to open peer review. As part of this commitment we make the peer review history of every article we publish publicly available.

When an article is published we post the peer reviewers' comments and the authors' responses online. We also post the versions of the paper that were used during peer review. These are the versions that the peer review comments apply to.

The versions of the paper that follow are the versions that were submitted during the peer review process. They are not the versions of record or the final published versions. They should not be cited or distributed as the published version of this manuscript.

BMJ Open is an open access journal and the full, final, typeset and author-corrected version of record of the manuscript is available on our site with no access controls, subscription charges or pay-per-view fees (http://bmjopen.bmj.com).

If you have any questions on BMJ Open's open peer review process please email info.bmjopen@bmj.com

BMJ Open

Protocol for A Randomised Phase II study of Accelerated, Dose escalated, Sequential Chemo-radiotherapy in Non-Small Cell Lung Cancer (ADSCaN)

Journal:	*BMJ Open*
Manuscript ID	bmjopen-2017-019903
Article Type:	Protocol
Date Submitted by the Author:	24-Oct-2017
Complete List of Authors:	Hatton, Matthew; Weston Park Hospital, Department of Clinical Oncology<br>Lawless, Claire; University of Glasgow, Cancer Research UK Clinical Trials Unit, Institute of Cancer Sciences<br>Faivre-Finn, Corinne; The Christie NHS Foundation Trust, Radiotherapy Related Research; University of Manchester, Manchester Academic Health Science Centre, Institute of Cancer Sciences<br>Landau, David; Guy's & St. Thomas' NHS Trust, Guy's Hospital, Great Maze Pond<br>Lester, Jason; Velindre NHS Trust UK, Department of Clinical Oncology<br>Fenwick, John; Clatterbridge Cancer Centre NHS Foundation Trust, Dept of Physics<br>Simões, Rita; Mount Vernon Hospital, National Radiotherapy Trials Quality Assurance Group<br>McCartney, Elaine; University of Glasgow, Cancer Research UK Clinical Trials Unit, Institute of Cancer Sciences<br>Boyd, Kathleen; University of Glasgow, Health Economics & Health Technology Assessment<br>Haswell, Tom; National Cancer Research Institute Consumer Liaison Group, Angel Building<br>Shaw, Ann; University of Glasgow, Cancer Research UK Clinical Trials Unit, Institute of Cancer Sciences<br>Paul, James; University of Glasgow, Cancer Research UK Clinical Trials Unit
Keywords:	Non-Small Cell Lung Cancer, sequential, chemo-radiotherapy, RADIOTHERAPY

SCHOLARONE™
Manuscripts

Protocol for A Randomised Phase II study of **A**ccelerated, **D**ose escalated, **S**equential **C**hemo-r**a**diotherapy in **N**on-Small Cell Lung Cancer (ADSCaN)

*Matthew QF Hatton — Dept. of Clinical Oncology, Weston Park Hospital, Sheffield, UK S10 2SJ

Claire Lawless, — CRUK Clinical Trials Unit, Institute of Cancer Sciences, University of Glasgow, UK

Corinne Faivre-Finn — Division Cancer Sciences, University of Manchester, UK

David Landau — Guy's & St. Thomas' NHS Trust, Guy's Hospital, Great Maze Pond, London

Jason F Lester — Velindre Cancer Centre, Velindre Road, Whitechurch, CARDIFF, CF14 2TL

John Fenwick — Dept of Physics, Clatterbridge Cancer Centre, Clatterbridge Road, Bebington, Wirral, CH63 4JY

Rita Simões — National Radiotherapy Trials Quality Assurance (RTTQA) Group, Mount Vernon Hospital, Middlesex, UK

Elaine McCartney — CRUK Clinical Trials Unit, Institute of Cancer Sciences, University of Glasgow, UK

Kathleen Boyd — Health Economics & Health Technology Assessment, Institute of Health & Wellbeing, University of Glasgow, Glasgow, UK

Tom Haswell — National Cancer Research Institute Consumer Liaison Group, Angel Building, 407 St John Street, London EC1V 4AD

Ann Shaw — CRUK Clinical Trials Unit, Institute of Cancer Sciences, University of Glasgow, UK

James Paul — CRUK Clinical Trials Unit, Institute of Cancer Sciences, University of Glasgow, UK

*Corresponding Author — Dept. of Clinical Oncology, Whitham Rd, Sheffield S10, 2SJ

Tel 0114-226-5071 E-mail Matthew.Hatton@sth.nhs.uk

For peer review only - http://bmjopen.bmj.com/site/about/guidelines.xhtml

Abstract

Introduction

Lung cancer is the most common cause of cancer mortality in the UK and Non-Small Cell Lung Cancer (NSCLC) accounts for approximately 85% of all lung cancers. Most patients present with inoperable disease therefore radiotherapy plays a major role in treatment. However, the majority of patients are not suitable for the gold standard treatment (concurrent chemo-radiotherapy) due to performance status and comorbidities. Novel strategies integrating radiotherapy advances and radiobiological knowledge need to be evaluated in patients treated with sequential chemo-radiotherapy. Four separate dose escalation accelerated radiotherapy schedules have been completed in UK (CHART-ED[(1)], IDEAL-CRT[(2)], I-START[(3)] and Isotoxic IMRT[(4)]). This study will compare these schedules with a UK standard sequential chemo-radiotherapy schedule of 55Gy in 20 fractions over 4 weeks. As it would be impossible to test all schedules in a phase III study the aim is to use a combined randomized phase II screening/"pick the winner" approach to identify the best schedule to take into a randomised phase III study against conventionally fractionated radiotherapy.

Methods and Analysis

Suitable patients will have histologically/cytologically confirmed, stage III NSCLC and are able to undergo chemo-radiotherapy treatment. The study will recruit 360 patients; 120 on the standard arm and 60 on each experimental arm. Patients will complete 2-4 cycles of platinum based chemotherapy before being randomised to one of the radiotherapy schedules. The primary endpoint is progression-free survival, with overall survival, time to local-regional failure, toxicity and cost-effectiveness as secondary objectives.

Ethics and dissemination:

The study has received ethical approval (REC reference: 16/WS/0165) from the West of Scotland Research Ethics Committee 1. The trial is conducted in accordance with the Declaration of Helsinki and Good Clinical Practice (GCP). Trial results will be published in a peer-reviewed journal and presented internationally.

Trial registration number: ISRCTN47674500

Keywords: Non-Small Cell Lung Cancer, Sequential Chemo-Radiotherapy.

For peer review only - http://bmjopen.bmj.com/site/about/guidelines.xhtml

Strengths and limitations of this study

Strengths

- Radiotherapy delivered using advanced techniques, including 4D-CT and image-guided radiotherapy (IGRT). Intensity modulated radiotherapy(IMRT) is mandated in one arm and recommended in all others
- Robust quality assurance (QA) programme
- Randomised multicentre study
- innovative multiarm "pick the winner" design

Limitations

- Heterogeneity of the patient group
- Not all experimental arms are available at all sites, so although arms are randomised against control, they are not randomised against each other
- Final analysis relies on adjusting for between-centre differences in key patient characteristics in order to "pool" the control arm and achieve the target power

For peer review only

For peer review only - http://bmjopen.bmj.com/site/about/guidelines.xhtml

INTRODUCTION

Background

Lung cancer is the most common cause of cancer mortality in the UK. In 2014 there were 46,400 new cases and 35,900 deaths and non-Small Cell Lung Cancer(NSCLC) accounts for approximately 85% of these. Most cases are inoperable at presentation however many patients with stage I-III disease can be treated radically using radiotherapy(RT), although survival rates are disappointing. Successful local control has been found to correlate with improved survival[(5)]. Strategies to achieve improved local control rely on intensification of local anti-tumour effect though radiation sensitization, acceleration of RT schedule or dose-escalation. As most patients are not suitable for gold standard treatment of concurrent chemo-radiotherapy, novel strategies integrating RT technological advances and radiobiological knowledge need evaluating in patients treated with the alternative option of sequential chemo-radiotherapy. The UK strategy has been to develop separate escalation protocols for hyper-fractionated and once daily schedules(CHART-ED[(1)], IDEAL-CRT[(2)], I-START[(3)], and Isotoxic IMRT[(4)]). These studies have completed recruitment and the results of 2 have been published[(1,2)].

Dose Acceleration / Intensification

In the UK, Continuous Hyper-fractionated Accelerated Radiation Therapy (CHART) trial [(6)] intensified local treatment by accelerating the RT course. When compared to conventional fractionation, there was a 9% absolute improvement in 2-year survival (29% v 20%; p=0.004) which provides evidence that intensification of local treatment only results in increased overall survival (OS). This result is supported by meta-analysis[(7)] reporting an absolute benefit for hyper-fractionated or accelerated radiotherapy over conventional fractionations of around 3% at 5 years.

Further evidence supporting intensification of local treatment comes from trials showing a survival benefit of concurrent chemo-radiotherapy over sequential[(5)]. This was achieved by chemotherapy increasing local tumour cell-kill and improving local control while maintaining the systemic effect of chemotherapy.

RT Dose Escalation

Increasing the radiotherapy dose has been associated with seemingly improved local control and OS in early-phase studies. However, RTOG 0617[(8)] comparing 60Gy in 30# to 74Gy in 37# once-daily showed that local control and OS were poorer on the higher dose. Reasons for this are being debated but it is clear that increasing RT by 2Gy per fraction per day with an increase in overall treatment time will not improve outcomes.

Isotoxic Radiotherapy

The MAASTRO group pioneered the concept of 'isotoxic RT' allowing for individualized dose escalation using hyperfractionated accelerated RT based on predefined and Mean Lung Dose (MLD) in stage I–III patients. This showed with 3D-CRT delivered two times a day over 4 weeks that increasing the radiation dose to pre-

For peer review only - http://bmjopen.bmj.com/site/about/guidelines.xhtml

specified normal tissue dose constraints could lead to increased tumour-control probability with the same normal tissue complication probability[9]. Survival of these stage III patients, all of whom were treated sequentially, were comparable to results expected from concurrent chemo-radiotherapy with acceptable acute and late toxicity. Since this study reported, Intensity modulated radiotherapy (IMRT) techniques have become routine practice improving targeting of radiation doses while decreasing doses to lungs and other normal tissue. This gives the potential to use the isotoxic approach to escalate the dose delivered to the target increasing tumour control probability with the same normal tissue complication probability[10].

Dose Acceleration and Escalation

Techniques that avoid prolongation of overall treatment time are attractive as they reduce the impact of accelerated tumour clonogen proliferation, which becomes clinically relevant for NSCLC approximately 3-4 weeks after radiotherapy initiation. A meta-analysis[7] of altered fractionations(10 trials, 2,000 NSCLC patients) showed modified fractionation improved OS as compared with conventional schedules(hazard ratio[HR]=0.88, 95% CI [0.80-0.97]; p=0.009), resulting in an absolute benefit of 2.5% at 5 years. CHART(54Gy in 36# over 12 days) remains one of the more commonly used schedules for radical treatment of NSCLC in the UK along with daily fractionated schedules delivering 55Gy in 20# over 4 weeks or 60-66Gy in 30-33# over 6-6½ weeks[11]. In current UK practice both CHART and the 4-week schedule are deliverable, with 99% of patients completing treatment, with survival outcome matching that seen in the original CHART study and very low levels of grade III/IV toxicity[12]. However, survival rates for all three regimens are low, and could be improved by dose-intensification.

Radiobiological modeling suggests that dose-escalation to improve the Tumour Control Probability (TCP) is likely to be more effective if the overall treatment time is fixed rather than fixing the dose per fraction, and dose-escalation typically adds 1-2% local control for each 1% increase in dose[13]. Therefore, CRUK funded 4 parallel dose-escalation studies to intensify accelerated schedules used in the UK. The first, CHART-ED (ISRCTN 45918260)[1] reported no dose limiting toxicities with dose-escalation(using additional twice-daily fractions of 1.8Gy) to 64.8 Gy in 17 days. This dose increase has the potential to increase local tumour control by 30%. The IDEAL CRT trial(ISRCTN 12155469) evaluated dose-escalation up to 73Gy in 30# over 6 weeks schedule with dose-escalation calculated on an individual patient basis according to either lung or oesophageal radiation dose. The study has established 68Gy to 1cc oesophagus as the MTD to be taken forward into future trials[2]. Both CHART-ED and IDEAL CRT report encouraging 2-year survival figures: 52% and 65% respectively.

Following the confirmation of low toxicity with the original IDEAL CRT trial, a further accelerated schedule was investigated where overall treatment time was shortened from 6 to 5 weeks by treating with RT twice a day on one day/week (not on chemotherapy day). The MTD to 1cc esophagus was reduced to 65Gy and the maximum prescription dose allowed was reduced to 71Gy in 30# (over 5 weeks). This portion of the study recruited 36 patients with a minimum 6-month follow up and no excess toxicity. This schedule will be taken forward and given sequentially in ADSCaN.

For peer review only - http://bmjopen.bmj.com/site/about/guidelines.xhtml

The two remaining studies, I-START (ISRCTN 74841904)[3] which individualized the RT dose similarly to IDEAL CRT using a 20# schedule following sequential chemotherapy and Isotoxic IMRT(NCT01836692)[4], where patients were treated twice-daily over 4 weeks to a maximum of 79.2Gy using pre-specified normal tissue doses have completed recruitment and neither report excess toxicity (personal communication).

Most patients with stage III NCLC are not suitable for gold standard concurrent chemo-radiotherapy[14], and novel strategies that integrate RT technological advances and radiobiological knowledge need evaluating in patients treated with sequential chemo-radiotherapy. This study aims to provide a framework for further development by identifying which is the best accelerated schedule to take forward into a randomised phase III study against conventionally fractionated radiotherapy.

METHODS AND ANALYSIS

ADSCaN is a non-blinded multicentre phase II study sponsored by NHS Greater Glasgow and Clyde Health Board and co-ordinated by the Cancer Research UK Clinical Trials Unit(CRUK CTU) based at the University of Glasgow. It is registered on the ISRCTN clinical trials database(ISRCTN47674500) and funded by Cancer Research UK's Clinical Trials Awards and Advisory Committee(CTAAC, Funder Reference: C9759/A16604). Radiotherapy quality assurance(QA) is supported by the National Radiotherapy Trial Quality Assurance(RTTQA) group. The study is included in the National Institute for Health Research(NIHR) Clinical Research Network portfolio(ID 35624) and is being conducted in accordance with the Declaration of Helsinki and Good Clinical Practice(GCP).

We hypothesize that sequential chemo-radiotherapy using accelerated, dose-escalated radiotherapy will improve progression-free survival(PFS), OS, time to local-regional failure, toxicity and cost-effectiveness compared to conventionally fractionated sequential chemo-radiotherapy treatment. ADSCaN will take the 4 dose escalated accelerated sequential chemo-radiotherapy schedules into a randomized phase II comparison with a UK standard sequential chemo-radiotherapy using state-of-the art radiotherapy.

Setting

ADSCaN opened on 22 August 2017 and will recruit 360 patients with a histological/cytological proven NSCLC across 40 UK radiotherapy centres, a list of which can be found on the NIHR Clinical Research Network Portfolio. Patients will be stage III, PS 0–1 and unsuitable for concurrent chemo-radiotherapy. Individual centres will randomise patients between standard radiotherapy(55 Gy in 20#) and one or more experimental arms. All sites will offer the standard arm, but may choose which experimental arms they wish to use (Figure 1: Flow diagram).

Participant screening and selection

Patients deemed inoperable by the lung multidisciplinary team(MDT) and suitable for sequential chemo-radiotherapy are invited to participate and provided with a patient information sheet (PIS, online supplementary appendix 1). Mandatory pre-randomisation investigations include: lung function tests, contrast

For peer review only - http://bmjopen.bmj.com/site/about/guidelines.xhtml

CT scan of thorax/upper abdomen within 4 weeks prior to randomisation and fluorodeoxyglucose positron emission tomography(FDG-PET) with CT(or MRI) brain scan if patients have not these investigations prior to starting induction chemotherapy.

Inclusion Criteria

- Histologically/cytologically confirmed stage III NSCLC
- ECOG Performance status(PS) 0-2(PS 2 only included if the general condition is explained by the primary chemotherapy treatment according to the investigator
- Inoperable disease, unsuitable for concurrent chemo-radiotherapy, in the opinion of the treating oncologist
- Patients with complete response, partial response or stable disease on CT assessment after 2 cycles of platinum-based chemotherapy
- Aged ≥16
- Adequate PFT results: FEV1 and/or KCO≥40% of predicted.

Exclusion Criteria

- Previous/current malignant disease likely to interfere with the protocol treatment or comparisons
- Clinically significant interstitial lung disease

Informed consent

Eligibility is confirmed by a clinician and patients are given ≥24 hours to consider the PIS and ask questions prior to written informed consent being taken.

Randomisation

Randomisation occurs after completion of chemotherapy. Once a patient is deemed eligible for the study and has consented to participate, the CRUK CTU, Glasgow, is contacted to confirm eligibility, and subsequently the patient is randomised to one of the available ADSCaN arms offered on-site and issued with a participant identification number. Patient enrolment details are confirmed by email.

Minimisation incorporating a random factor is used to allocate patients between treatment arms A:B:C:D:E so that an overall study ratio of 2:1:1:1:1 is achieved. The following stratification factors are used:

- Hospital
- Performance Status
- Intention to use IMRT
- Histological type
- Stage
- EGFR mutation status
- Gross Tumour Volume(GTV)
- Haemoglobin
- Serum albumin
- Sex

For peer review only - http://bmjopen.bmj.com/site/about/guidelines.xhtml

- Weight loss since diagnosis

While on study, participants will not co-enrol in other clinical trials offering therapeutic intervention.

Standard care

In patients with inoperable stage III NSCLC who are unsuitable for concurrent chemo-radiotherapy, sequential chemo-radiotherapy is the standard of care.

RT intervention and planning

4D-CT with intravenous contrast should be used for all ADSCaN patients to account for tumour motion during breathing. The whole thorax(cricoid to L2) should be covered to allow dose volume histograms to be calculated for the lung, heart, spinal cord, brachial plexus, great vessels, proximal bronchial tree and the oesophagus.

The input of a thoracic radiologist is recommended when defining the identifiable tumour and involved lymph nodes to give the GTV. The thoracic radiologist should work with a qualified radiation oncologist specialised in thoracic malignancies to produce a motion adapted GTV.

The 4D-GTV is expanded by 5mm to create the Clinical Target Volume(CTV). The CTV will be expanded to include microscopic spread and may be edited to account for anatomical barriers but only if it is not thought to invade the structure.

CTV to planning target volume(PTV) expansion takes into account patient set-up uncertainties.

- If a centre is using per fraction Cone Beam CT(CBCT) imaging and online correction for treatment verification, the CTV should be expanded by 5mm isotropically to create the PTV.
- If a centre is not CBCT imaging per fraction, the CTV should be expanded by 7mm axially and 9mm cranio/caudally to create the PTV.

Prescribed dose and fractionation

Radiotherapy schedules should begin on a Monday where possible to reduce overall treatment time. Schedules are summarised in Table 1 and dose constraints for organs at risk in Table 2.

All patients recruited at a centre must be treated with the same planning technique irrespective of randomisation arm. The use of IMRT is strongly recommended(and mandated for Arm E) using 5 or more fields in an IMRT plan or an arc technique may be used.

Follow-up

Patients are followed up monthly for 3 months, then 3-monthly to 2 years, 6-monthly to 3 years then annually. A late toxicity assessment will be performed at each visit.

Withdrawal

Patients can withdraw from the trial at any time without any effect on clinical care.

Quality of Life

For peer review only - http://bmjopen.bmj.com/site/about/guidelines.xhtml

Quality of life(QoL) as measured by EQ-5D will be completed at all study visits and weekly during treatment.

Data Collection

All information collected during the course of the study will be kept strictly confidential. Data will be entered on to an electronic case report form system with built-in edit checks to ensure high quality data. Sites will be monitored centrally by checking incoming data for compliance with the protocol, data consistency, missing data and timing.

Statistical considerations

The comparison of each experimental arm to standard treatment has 80% power to detect a 33% increase in median PFS(from 8 to 10.7 months) at the 20% 1-sided level of statistical significance. A 33% improvement in PFS was selected as it was felt realistic and that an improvement of this magnitude was required to ensure UK support for any subsequent phase III. There is no formal calculation of sample size for the "pick-the-winner" element; if more than one experimental arm passes the screening threshold a choice will be made using efficacy, toxicity, QoL and health economics (HE) evidence.

Primary Analysis

The primary statistical analysis will be based around a Cox model fitted to PFS incorporating terms for study arm and other prognostic factors that reflect case-mix. Model fit and the proportional hazards assumption will be checked by plotting Schoenfeld residuals and a test for goodness of fit[(15)]. The assessment of the effect of an experimental arm relative to standard and experimental arms relative to one another will be made via the hazard ratios(and associated p-values) derived from this model.

Secondary Analysis

The analysis of OS and time to local-regional failure will be analysed as for PFS.

Toxicity will be assessed using NCI CTCAE v4.03. Late effects will be monitored. The worst grade will summarized and compared between study arms using the Mann-Whitney U-test. HE analysis will be undertaken from the perspective of the UK National Health Service(NHS), adhering to the National Institute for Health Care Excellence(NICE) reference case to assess cost-effectiveness of the experimental arms compared to current standard. QoL will be assessed using EQ-5D and parametric extrapolation undertaken on the PFS and OS curves to generate lifetime outcomes and compare across study arms in terms of quality adjusted life year(QALY) gains.

Interim Analysis

Study data will be reviewed at least annually by an Independent Data Monitoring Committee (IDMC). Safety stopping in the experimental arms will be monitored continuously based on grade 4/5 acute radiation toxicity.

Changes to the protocol after the start of the trial

For peer review only - http://bmjopen.bmj.com/site/about/guidelines.xhtml

This document is consistent with ADSCaN study protocol(Version 1.2, dated 20th January 2017). There have been no significant changes to the protocol since the start of the study.

End of the trial

The study will close 12 months after the last patient has recruited or when 305 PFS events have been observed (whichever is later). The chief investigator and/or the trial management group (TMG) have the right at any time to terminate the study for clinical or administrative reasons. The end of the study will be reported to the REC and Regulatory Authority (where applicable) within the required timeframes.

Quality Assurance programme

The trial is subject to a RT QA programme, which includes pre-trial and on-trial components. Dosimetry audit will be performed in centres where a recent relevant external audit has not been completed. Every attempt will be made to streamline with previous national and international audits. The QA programme for the study is coordinated by the UK RTTQA Group. The details of the programme can be found at the RTTQA website, http://www.rttrialsqa.org.uk.

The pre-trial QA includes:

- Facility questionnaire.
- Outlining benchmark case. Target volumes and organs at risk(OAR) are delineated on a CT data set provided. The OAR should be outlined according to the ADSCaN OAR Atlas.
- Planning benchmark case. Planned according to the trial protocol on a pre-outlined CT(GTV, CTV, PTV and OAR).

Centres that have previously completed pre-trial QA programmes for the individual trial arms, CHART-ED, IDEAL, I-START and Isotoxic IMRT, may be eligible for QA streamlining.

The on-trial QA includes Individual Case Reviews (ICR) to monitor protocol adherence:

- Prospective ICR for at least the first patient from each centre for each trial arm. Retrospective ICR for selected cases.

ETHICS AND DISSEMINATION

Adverse events are collected (according to the CTCAE V.4.0 grading system) at each trial visit and all adverse events on study are reported to the CRUK CTU Glasgow and followed until they resolve or stabilise. Acute and late radiation toxicities continue to be recorded at each follow-up visit.

Trial monitoring and oversight

Formal on site data monitoring activities are performed as part of the ADSCaN study. Each site will receive telephone and on-site monitoring visits during the course of the study.

For peer review only - http://bmjopen.bmj.com/site/about/guidelines.xhtml

Data is reviewed by an IDMC (comprising two clinicians and one statistician) and Trials Steering Committee. The TMG coordinates and manages the trial's day-to-day activities and is comprised of health professionals, a patient representative and members of the direct study team, including the principal investigators for each of the experimental arms.

Dissemination

Data from all centres will be analysed together and published promptly. Individual participants may not publish data concerning their patients that are directly relevant to questions posed by the trial until the TMG has published its report. The TMG will form the basis of the Writing Committee and advise on publications. The trial will be publicised at regional and national conferences. The final results will be presented at scientific meetings and published in a peer-reviewed journal(authorship will be according to the journal's guidelines). In addition a lay summary of the results will be produced for interested parties for example, Cancer Research UK. The CTU is committed to furthering research by sharing de-identified individual-patient data(IPD) from its studies with others in the field who wish to use the data for high quality science and we are happy to consider proposals from researchers and will share IPD to the maximum extent.

For peer review only

For peer review only - http://bmjopen.bmj.com/site/about/guidelines.xhtml

Trial status

The study opened to recruitment on 22nd August 2017.

Author contributions

MH conceived of the study. MH, CF-F, DL, JL, JF, JP, EM, JM, KB initiated the study design and CL, RS, AP and AS helped with implementation. MH is the grant holder. JP provided statistical expertise in clinical trial design. All the authors contributed to refinement of the study protocol and approved the final manuscript. TH is the patient representative inputting into study design and Implementation.

Acknowledgements The authors would like to acknowledge the support of the study sponsor NHS Greater Glasgow & Clyde, study funder CRUK and the patients who consent to enter the study. In addition we would like to acknowledge the individual contributions of Joe Maguire, The Clatterbridge Cancer Centre, Wirral, Emma Parsons, Elizabeth Miles(National Radiotherapy Trials Quality Assurance(RTTQA) Group, Mount Vernon Hospital, Middlesex, UK), Joanne McGarry(NHS Greater Glasgow & Clyde), Stephen Clark, Lindsey Connery, Calum Innes(CRUK Clinical Trials Unit, Institute of Cancer Sciences, University of Glasgow, UK).

Patient and carer representatives have been involved in the development of ADSCaN through the National Cancer Research Institute(NCRI) Lung Clinical Studies Group(CSG) and the West of Scotland Cancer Research Network Consumer Research Panel. In Particular, we thank Tom Haswell our consumer representative is part of the Trial Management Group. He commented on the preparation of the final protocol, Research Ethics application and patient information sheet and consent form and this manuscript.

Contributors All the authors contributed to refinement the study protocol and approved the final manuscript.

Funding This research is funded by Cancer Research UK's(CRUK) Clinical Trials Awards and Advisory Committee(CTAAC). Grant reference number A16604.

Competing interests None declared.

Ethics approval West of Scotland Research Ethics Committee, West of Scotland REC 1, 8th November 2016 (REC reference: 16/WS/0165)

Sponsor: NHS Greater Glasgow and Clyde(Greater Glasgow Health Board: J B Russell House, Gartnavel Royal Hospital, 1055 Great Western Road, Glasgow, G12 0XH)

The Sponsor is responsible for confirming there are proper arrangements for the initiation and management of the study. The majority of the Sponsor's responsibilities have been delegated to the Chief Investigator(CI) who performs these via the CRUK CTU as the co-ordinating centre for the trial. As such, the main role of the

For peer review only - http://bmjopen.bmj.com/site/about/guidelines.xhtml

Sponsor is to ensure that the CI and CRUK CTU fulfil their responsibilities as outlined in the Clinical Trial Agreement and to ensure that any identified “risks” either have controls or action points put in place.

For peer review only

For peer review only - http://bmjopen.bmj.com/site/about/guidelines.xhtml

Figure 1 ADSCaN Trial Schema

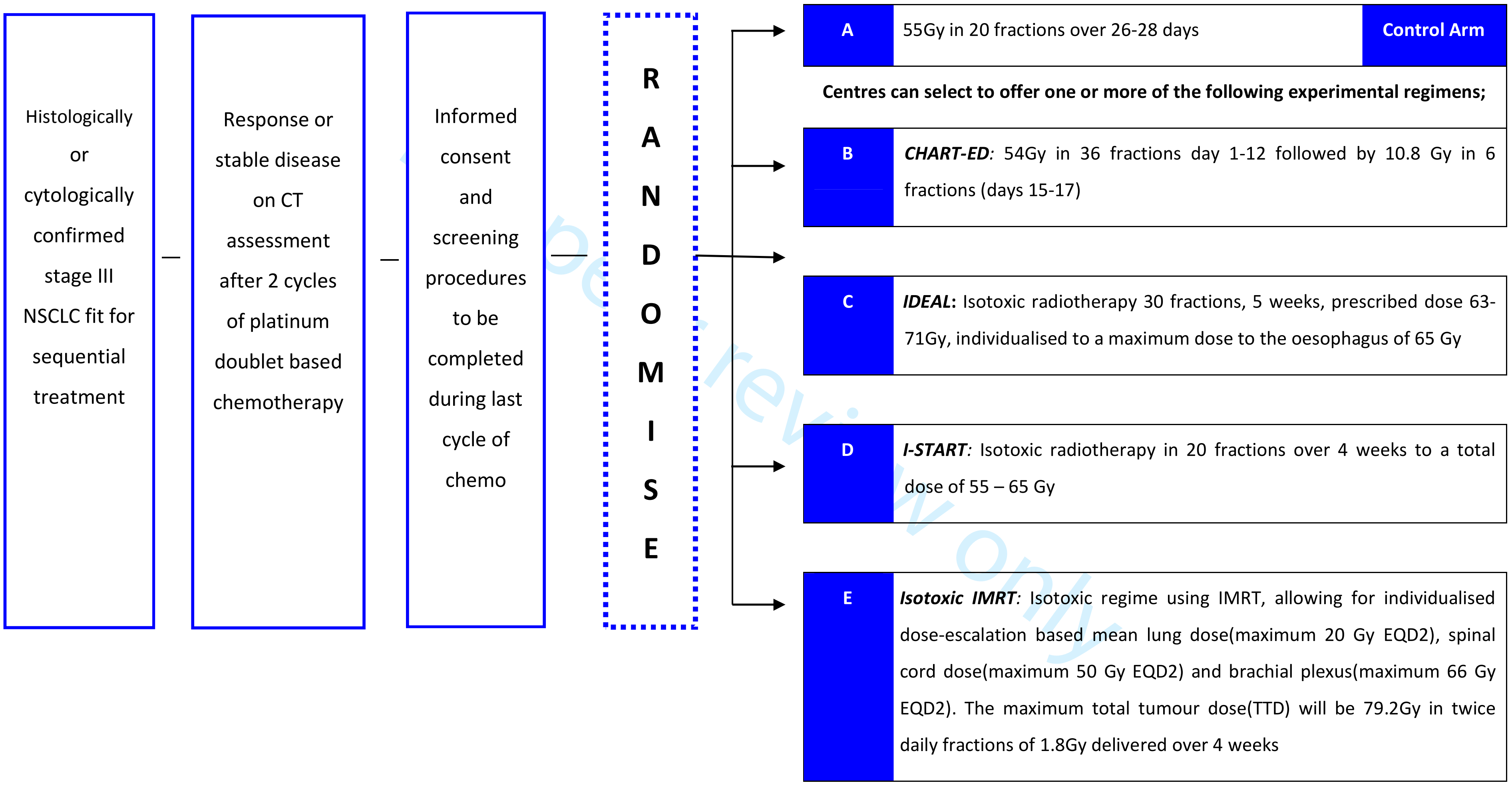

For peer review only - http://bmjopen.bmj.com/site/about/guidelines.xhtml

Table 1: Summary of Trial Arms

Trial arm	Total dose	Total fractions	fractions per day	Total number of days
**Arm A:** Standard Arm	55.0Gy	20	1	26-28
**Arm B:** CHART-ED	54.0Gy<br>10.8Gy	36<br>6	3<br>2	Days 1-12<br>Days 15-17
**Arm C:** IDEAL	63-71Gy	30	1 with 1 B.D treatment per week	33-35
**Arm D:** I-START	55-65Gy	20	1	26-28
**Arm E:** Isotoxic IMRT	61.2-79.2Gy	34-44	2	23-30 depending on dose-escalation

For peer review only

For peer review only - http://bmjopen.bmj.com/site/about/guidelines.xhtml

Table 2: Dose constraints for all trial arms

Volume	Arm A: Standard	Arm B: CHART-ED	Arm C: IDEAL-CRT	Arm D: I-START	Arm E: Isotoxic IMRT
CTV	V95% >99%	V95% > 99%	V95% > 99%	V95% > 99%	Optimal: CTV $D_{(Total\ Vol\ -1cc)}$ ≥95%<br>Mandatory: $CTV^{MeanDose}$+/- 1% of prescribed dose
PTV	V95% > 90%<br>V90% >98%	V95% > 90%<br>V90% > 98%	V95% > 90%<br>V90% > 98%	V95% > 90%<br>V90% > 98%	Optimal: PTV $D_{(Total\ Vol\ -1cc)}$ ≥90%<br>PTV D95% ≥95%<br>Mandatory: PTV $D_{(Total\ Vol-1cc)}$ ≥85%<br>PTV D95% ≥90%<br>PTV D1cc ≤107%
Spinal Canal	D0.1cc <47Gy	D0.1cc <46Gy	D0.1cc < 48Gy	D0.1cc < 47Gy	D1.0cc ≤ EQD2 50Gy
Lung-GTV	V20Gy <35%	V20Gy <35%	V20Gy < 35%		Mean Dose ≤20Gy(physical dose)
Brachial Plexus	D0.1cc <55Gy	D0.1cc <64.3Gy	D0.1cc < 65Gy<br>D30% < 60Gy(Ipsilateral BPlexus)	D0.1cc <55Gy	D1.0cc ≤ EQD2 66Gy
Heart	D100% < 36Gy<br>D67% < 44Gy<br>D33% < 57Gy	D100% < 48Gy<br>D67% <57Gy	D100% < 44Gy<br>D67% < 52Gy<br>D33% < 59Gy	D100% < 36Gy<br>D67% < 44Gy<br>D33% < 57Gy	D1.0cc ≤ EQD2 76Gy<br>Mean Dose ≤46Gy(physical dose)
Oesophagus	D0.1cc <105%	D0.1cc <105%	D1.0cc < 65y		
Lung Contra	V5Gy <60%*		V5Gy <60% *	V5Gy < 60%*	
Mediastinal Env					D1.0cc ≤ EQD2 76Gy

* Optimal constraint (i.e. not mandatory limit)

For peer review only

For peer review only - http://bmjopen.bmj.com/site/about/guidelines.xhtml

REFERENCES

1. Hatton M, Hill R, Wilson P, Atherton P, Morgan S, Dickson J, Murray K, Paul J: Continuous Hyperfractionated Accelerated RadioTherapy – Escalated Dose(CHART-ED): A Phase I study: Radiotherapy Oncology. 2016;118:471-77. ISRCTN: 45918260
2. Landau DB, Khan I, Baker A et.al. IDEAL-CRT: A Phase 1/2 Trial of Isotoxic Dose-Escalated Radiation Therapy and Concurrent Chemotherapy in Patients with Stage II/III Non-Small Cell Lung Cancer. Int J Radiat Oncol Biol Phys 2016; 95:1367-1377 ISRCTN: 12155469
3. I-START Study ISRCTN: 74841904
4. Haslett K, Franks K, Hanna GG, Harden S, Hatton M, Harrow S et.al. Protocol for the isotoxic intensity modulated radiotherapy(IMRT) in stage III non-small cell lung cancer(NSCLC): a feasibility study. BMJ Open 2016;6:e010457.doi:10.1136/bmjopen-2015-010457
5. Aupérin A, Le Péchoux C, Rolland E, Curran W, Furuse K, Fourne P et.al. : Meta-analysis of concomitant versus sequential radio-chemotherapy in locally advanced non-small-cell lung cancer. J Clin Oncol 2010;28(13):2181-90
6. Saunders M, Dische S, Barrett A et al., for the CHART Steering Committee. Continuous hyperfractionated accelerated radio-therapy(CHART) versus conventional radiotherapy in non-small-cell lung cancer: A randomised multicentre trial. Lancet 1997; 350:161–165.
7. Mauguen A, Le Péchoux C, Saunders MI, Schild SE, Turrisi, Baumann M, et.al. Hyperfractionated or Accelerated Radiotherapy in Lung Cancer: An Individual Patient Data Meta-Analysis. J Clin Oncol. 2010; 28:2181-90
8. Bradley JD, Paulus R, Komaki R et al. Standard-dose versus high-dose conformal radiotherapy with concurrent and consolidation carboplatin plus paclitaxel with or without cetuximab for patients with stage IIIA or IIIB non-small-cell lung cancer(RTOG 0617): a randomised, two-by-two factorial phase 3 study. Lancet Oncol 2015; 16(2):187-99
9. van Baardwijk A, Wanders S, Boersma L, Borger J, Oellers M, Dingemans AMC, et.al. Mature results of an individualized radiation dose prescription study based on normal tissue constraints in stages I to III non-small-cell lung cancer. J Clin Oncol 2010; 28(8):1380-6
10. Chan C, Lang S, Rowbottom C, Guckenberger M, Faivre-Finn C; IASLC Advanced Radiation Technology Committee. Intensity-modulated radiotherapy for lung cancer: current status and future developments. J Thorac Oncol. 2014;9(11):1598-608.
11. Helbrow J, Bayman N, MacNicoll F, Faivre-Finn C: Concurrent chemo-radiotherapy(cCTRT) for locally advanced unresectable non-small cell lung cancer(LA-NSCLC): A national survey of current practice. Lung Cancer 2012; 75(1):S50-S51
12. Pemberton LS, Din OS, Fisher PM, Hatton MQ. Accelerated radical radiotherapy for non-small cell lung cancer(NSCLC) using two common regimens: a single centre audit of outcome. Clinical Oncology 2009;21:161-7
13. Fenwick JD, Nahum AE, Malik MI et al. Escalation and Intensification of Radiotherapy for Stage III Non-small Cell Lung Cancer: Opportunities for Treatment Improvement. Clinical Oncology 21:343-360;2009
14. De Ruysscher D, Botterweck A, Dirx M et al. Eligibility for concurrent chemotherapy and radiotherapy of locally advanced lung cancer patients: a prospective, population-based study. Ann Oncol 2009;(1):98-102
15. Parzen, M; Lipsitz, SR: A global goodness-of-fit statistic for Cox regression models BIOMETRICS Volume: 55 Issue: 2 Pages: 580-584: JUN 1999

For peer review only - http://bmjopen.bmj.com/site/about/guidelines.xhtml

SPIRIT

STANDARD PROTOCOL ITEMS: RECOMMENDATIONS FOR INTERVENTIONAL TRIALS

SPIRIT 2013 Checklist: Recommended items to address in a clinical trial protocol and related documents*

Section/item	Item No	Description	Addressed on page number
**Administrative information**			
Title	1	Descriptive title identifying the study design, population, interventions, and, if applicable, trial acronym	1
Trial registration	2a	Trial identifier and registry name. If not yet registered, name of intended registry	2
	2b	All items from the World Health Organization Trial Registration Data Set	NA
Protocol version	3	Date and version identifier	9
Funding	4	Sources and types of financial, material, and other support	12
Roles and responsibilities	5a	Names, affiliations, and roles of protocol contributors	1, 12
	5b	Name and contact information for the trial sponsor	12
	5c	Role of study sponsor and funders, if any, in study design; collection, management, analysis, and interpretation of data; writing of the report; and the decision to submit the report for publication, including whether they will have ultimate authority over any of these activities	12
	5d	Composition, roles, and responsibilities of the coordinating centre, steering committee, endpoint adjudication committee, data management team, and other individuals or groups overseeing the trial, if applicable (see Item 21a for data monitoring committee)	10,12

For peer review only - http://bmjopen.bmj.com/site/about/guidelines.xhtml

**Introduction**			
Background and rationale	6a	Description of research question and justification for undertaking the trial, including summary of relevant studies (published and unpublished) examining benefits and harms for each intervention	3
	6b	Explanation for choice of comparators	3-5
Objectives	7	Specific objectives or hypotheses	5
Trial design	8	Description of trial design including type of trial (eg, parallel group, crossover, factorial, single group), allocation ratio, and framework (eg, superiority, equivalence, noninferiority, exploratory)	5
**Methods: Participants, interventions, and outcomes**			
Study setting	9	Description of study settings (eg, community clinic, academic hospital) and list of countries where data will be collected. Reference to where list of study sites can be obtained	6
Eligibility criteria	10	Inclusion and exclusion criteria for participants. If applicable, eligibility criteria for study centres and individuals who will perform the interventions (eg, surgeons, psychotherapists)	7
Interventions	11a	Interventions for each group with sufficient detail to allow replication, including how and when they will be administered	13
	11b	Criteria for discontinuing or modifying allocated interventions for a given trial participant (eg, drug dose change in response to harms, participant request, or improving/worsening disease)	7, 8
	11c	Strategies to improve adherence to intervention protocols, and any procedures for monitoring adherence (eg, drug tablet return, laboratory tests)	NA
	11d	Relevant concomitant care and interventions that are permitted or prohibited during the trial	NA
Outcomes	12	Primary, secondary, and other outcomes, including the specific measurement variable (eg, systolic blood pressure), analysis metric (eg, change from baseline, final value, time to event), method of aggregation (eg, median, proportion), and time point for each outcome. Explanation of the clinical relevance of chosen efficacy and harm outcomes is strongly recommended	5
Participant timeline	13	Time schedule of enrolment, interventions (including any run-ins and washouts), assessments, and visits for participants. A schematic diagram is highly recommended (see Figure)	8

For peer review only - http://bmjopen.bmj.com/site/about/guidelines.xhtml

Sample size	14	Estimated number of participants needed to achieve study objectives and how it was determined, including clinical and statistical assumptions supporting any sample size calculations	5, 9
Recruitment	15	Strategies for achieving adequate participant enrolment to reach target sample size	3
**Methods: Assignment of interventions (for controlled trials)**			
Allocation:			
Sequence generation	16a	Method of generating the allocation sequence (eg, computer-generated random numbers), and list of any factors for stratification. To reduce predictability of a random sequence, details of any planned restriction (eg, blocking) should be provided in a separate document that is unavailable to those who enrol participants or assign interventions	7
Allocation concealment mechanism	16b	Mechanism of implementing the allocation sequence (eg, central telephone; sequentially numbered, opaque, sealed envelopes), describing any steps to conceal the sequence until interventions are assigned	7
Implementation	16c	Who will generate the allocation sequence, who will enrol participants, and who will assign participants to interventions	7
Blinding (masking)	17a	Who will be blinded after assignment to interventions (eg, trial participants, care providers, outcome assessors, data analysts), and how	NA
	17b	If blinded, circumstances under which unblinding is permissible, and procedure for revealing a participant's allocated intervention during the trial	NA
**Methods: Data collection, management, and analysis**			
Data collection methods	18a	Plans for assessment and collection of outcome, baseline, and other trial data, including any related processes to promote data quality (eg, duplicate measurements, training of assessors) and a description of study instruments (eg, questionnaires, laboratory tests) along with their reliability and validity, if known. Reference to where data collection forms can be found, if not in the protocol	9
	18b	Plans to promote participant retention and complete follow-up, including list of any outcome data to be collected for participants who discontinue or deviate from intervention protocols	8

For peer review only - http://bmjopen.bmj.com/site/about/guidelines.xhtml

Data management	19	Plans for data entry, coding, security, and storage, including any related processes to promote data quality (eg, double data entry; range checks for data values). Reference to where details of data management procedures can be found, if not in the protocol	9
Statistical methods	20a	Statistical methods for analysing primary and secondary outcomes. Reference to where other details of the statistical analysis plan can be found, if not in the protocol	9
	20b	Methods for any additional analyses (eg, subgroup and adjusted analyses)	NA
	20c	Definition of analysis population relating to protocol non-adherence (eg, as randomised analysis), and any statistical methods to handle missing data (eg, multiple imputation)	NA
**Methods: Monitoring**			
Data monitoring	21a	Composition of data monitoring committee (DMC); summary of its role and reporting structure; statement of whether it is independent from the sponsor and competing interests; and reference to where further details about its charter can be found, if not in the protocol. Alternatively, an explanation of why a DMC is not needed	10
	21b	Description of any interim analyses and stopping guidelines, including who will have access to these interim results and make the final decision to terminate the trial	8
Harms	22	Plans for collecting, assessing, reporting, and managing solicited and spontaneously reported adverse events and other unintended effects of trial interventions or trial conduct	10
Auditing	23	Frequency and procedures for auditing trial conduct, if any, and whether the process will be independent from investigators and the sponsor	10
**Ethics and dissemination**			
Research ethics approval	24	Plans for seeking research ethics committee/institutional review board (REC/IRB) approval	2, 12
Protocol amendments	25	Plans for communicating important protocol modifications (eg, changes to eligibility criteria, outcomes, analyses) to relevant parties (eg, investigators, REC/IRBs, trial participants, trial registries, journals, regulators)	NA

For peer review only - http://bmjopen.bmj.com/site/about/guidelines.xhtml

Consent or assent	26a	Who will obtain informed consent or assent from potential trial participants or authorised surrogates, and how (see Item 32)	6
	26b	Additional consent provisions for collection and use of participant data and biological specimens in ancillary studies, if applicable	NA
Confidentiality	27	How personal information about potential and enrolled participants will be collected, shared, and maintained in order to protect confidentiality before, during, and after the trial	
Declaration of interests	28	Financial and other competing interests for principal investigators for the overall trial and each study site	10
Access to data	29	Statement of who will have access to the final trial dataset, and disclosure of contractual agreements that limit such access for investigators	9
Ancillary and post-trial care	30	Provisions, if any, for ancillary and post-trial care, and for compensation to those who suffer harm from trial participation	NA
Dissemination policy	31a	Plans for investigators and sponsor to communicate trial results to participants, healthcare professionals, the public, and other relevant groups (eg, via publication, reporting in results databases, or other data sharing arrangements), including any publication restrictions	9
	31b	Authorship eligibility guidelines and any intended use of professional writers	NA
	31c	Plans, if any, for granting public access to the full protocol, participant-level dataset, and statistical code	11
**Appendices**			
Informed consent materials	32	Model consent form and other related documentation given to participants and authorised surrogates	Appendix
Biological specimens	33	Plans for collection, laboratory evaluation, and storage of biological specimens for genetic or molecular analysis in the current trial and for future use in ancillary studies, if applicable	NA

*It is strongly recommended that this checklist be read in conjunction with the SPIRIT 2013 Explanation & Elaboration for important clarification on the items. Amendments to the protocol should be tracked and dated. The SPIRIT checklist is copyrighted by the SPIRIT Group under the Creative Commons "Attribution-NonCommercial-NoDerivs 3.0 Unported" license.

For peer review only - http://bmjopen.bmj.com/site/about/guidelines.xhtml

BMJ Open

Protocol for A Randomised Phase II study of Accelerated, Dose escalated, Sequential Chemo-radiotherapy in Non-Small Cell Lung Cancer (ADSCaN)

Journal:	*BMJ Open*
Manuscript ID	bmjopen-2017-019903.R1
Article Type:	Protocol
Date Submitted by the Author:	23-Jul-2018
Complete List of Authors:	Hatton, Matthew; Weston Park Hospital, Department of Clinical Oncology<br>Lawless, Claire; University of Glasgow, Cancer Research UK Clinical Trials Unit, Institute of Cancer Sciences<br>Faivre-Finn, Corinne; The Christie NHS Foundation Trust, Radiotherapy Related Research; University of Manchester, Manchester Academic Health Science Centre, Institute of Cancer Sciences<br>Landau, David; Guy's & St. Thomas' NHS Trust, Guy's Hospital, Great Maze Pond<br>Lester, Jason; Velindre NHS Trust UK, Department of Clinical Oncology<br>Fenwick, John; Clatterbridge Cancer Centre NHS Foundation Trust, Dept of Physics<br>Simões, Rita; Mount Vernon Hospital, National Radiotherapy Trials Quality Assurance Group<br>McCartney, Elaine; University of Glasgow, Cancer Research UK Clinical Trials Unit, Institute of Cancer Sciences<br>Boyd, Kathleen; University of Glasgow, Health Economics & Health Technology Assessment<br>Haswell, Tom; National Cancer Research Institute Consumer Liaison Group, Angel Building<br>Shaw, Ann; University of Glasgow, Cancer Research UK Clinical Trials Unit, Institute of Cancer Sciences<br>Paul, James; University of Glasgow, Cancer Research UK Clinical Trials Unit
**Primary Subject Heading**:	Oncology
Secondary Subject Heading:	Respiratory medicine, Radiology and imaging, Evidence based practice, Health economics
Keywords:	Non-Small Cell Lung Cancer, sequential, chemo-radiotherapy, RADIOTHERAPY

SCHOLARONE™
Manuscripts

For peer review only - http://bmjopen.bmj.com/site/about/guidelines.xhtml

Protocol for A Randomised Phase II study of **A**ccelerated, **D**ose escalated, **S**equential **C**hemo-r**a**diotherapy in **N**on-Small Cell Lung Cancer (ADSCaN)

*Matthew QF Hatton — Dept. of Clinical Oncology, Weston Park Hospital, Sheffield, UK S10 2SJ

Claire Lawless, — CRUK Clinical Trials Unit, Institute of Cancer Sciences, University of Glasgow, UK

Corinne Faivre-Finn — Division Cancer Sciences, University of Manchester, UK

David Landau — Guy's & St. Thomas' NHS Trust, Guy's Hospital, Great Maze Pond, London

Jason F Lester — Velindre Cancer Centre, Velindre Road, Whitechurch, CARDIFF, CF14 2TL

John Fenwick — Dept of Physics, Clatterbridge Cancer Centre, Clatterbridge Road, Bebington, Wirral, CH63 4JY

Rita Simões — National Radiotherapy Trials Quality Assurance (RTTQA) Group, Mount Vernon Hospital, Middlesex, UK

Elaine McCartney — CRUK Clinical Trials Unit, Institute of Cancer Sciences, University of Glasgow, UK

Kathleen Boyd — Health Economics & Health Technology Assessment, Institute of Health & Wellbeing, University of Glasgow, Glasgow, UK

Tom Haswell — National Cancer Research Institute Consumer Liaison Group, Angel Building, 407 St John Street, London EC1V 4AD

Ann Shaw — CRUK Clinical Trials Unit, Institute of Cancer Sciences, University of Glasgow, UK

James Paul — CRUK Clinical Trials Unit, Institute of Cancer Sciences, University of Glasgow, UK

*Corresponding Author — Dept. of Clinical Oncology, Whitham Rd, Sheffield S10, 2SJ
Tel 0114-226-5071 E-mail Matthew.Hatton@sth.nhs.uk

For peer review only - http://bmjopen.bmj.com/site/about/guidelines.xhtml

Abstract

Introduction

Lung cancer is the most common cause of cancer mortality in the UK and Non-Small Cell Lung Cancer (NSCLC) accounts for approximately 85% of all lung cancers. Most patients present with inoperable disease therefore radiotherapy plays a major role in treatment. However, the majority of patients are not suitable for the gold standard treatment (concurrent chemo-radiotherapy) due to performance status and comorbidities. Novel strategies integrating radiotherapy advances and radiobiological knowledge need to be evaluated in patients treated with sequential chemo-radiotherapy. Four separate dose escalation accelerated radiotherapy schedules have been completed in UK (CHART-ED, IDEAL-CRT, I-START and Isotoxic IMRT). This study will compare these schedules with a UK standard sequential chemo-radiotherapy schedule of 55Gy in 20 fractions over 4 weeks. As it would be impossible to test all schedules in a phase III study the aim is to use a combined randomized phase II screening/"pick the winner" approach to identify the best schedule to take into a randomised phase III study against conventionally fractionated radiotherapy.

Methods and Analysis

Suitable patients will have histologically/cytologically confirmed, stage III NSCLC and are able to undergo chemo-radiotherapy treatment. The study will recruit 360 patients; 120 on the standard arm and 60 on each experimental arm. Patients will complete 2-4 cycles of platinum based chemotherapy before being randomised to one of the radiotherapy schedules. The primary endpoint is progression-free survival, with overall survival, time to local-regional failure, toxicity and cost-effectiveness as secondary objectives.

Ethics and dissemination:

The study has received ethical approval (REC reference: 16/WS/0165) from the West of Scotland Research Ethics Committee 1. The trial is conducted in accordance with the Declaration of Helsinki and Good Clinical Practice (GCP). Trial results will be published in a peer-reviewed journal and presented internationally.

Trial registration number: ISRCTN47674500

Keywords: Non-Small Cell Lung Cancer, Sequential Chemo-Radiotherapy.

For peer review only

For peer review only - http://bmjopen.bmj.com/site/about/guidelines.xhtml

Strengths and limitations of this study

Strengths

- Radiotherapy delivered using advanced techniques, including 4D-CT and image-guided radiotherapy (IGRT). Intensity modulated radiotherapy(IMRT) is mandated in one arm and recommended in all others
- Robust quality assurance (QA) programme
- innovative multiarm “pick the winner” design

Limitations

- Not all experimental arms are available at all sites, so although arms are randomised against control, they are not randomised against each other
- Final analysis relies on adjusting for between-centre differences in key patient characteristics in order to “pool” the control arm and achieve the target power

For peer review only

For peer review only - http://bmjopen.bmj.com/site/about/guidelines.xhtml

INTRODUCTION

Background

Lung cancer is the most common cause of cancer mortality in the UK. In 2014 there were 46,400 new cases and 35,900 deaths and non-Small Cell Lung Cancer(NSCLC) accounts for approximately 85% of these. Most cases are inoperable at presentation however many patients with stage I-III disease can be treated radically using radiotherapy(RT), although survival rates are disappointing. Successful local control has been found to correlate with improved survival[(1)]. Strategies to achieve improved local control rely on intensification of local anti-tumour effect though radiation sensitization, acceleration of RT schedule or dose-escalation. As most patients are not suitable for gold standard treatment of concurrent chemo-radiotherapy, novel strategies integrating RT technological advances and radiobiological knowledge need evaluating in patients treated with the alternative option of sequential chemo-radiotherapy. The UK strategy has been to develop separate escalation protocols for hyper-fractionated and once daily schedules(CHART-ED[(2)], IDEAL-CRT[(3)], I-START[(4)], and Isotoxic IMRT[(5)]). These studies have completed recruitment and the results of 2 have been published[(1,2)].

Dose Acceleration / Intensification

In the UK, Continuous Hyper-fractionated Accelerated Radiation Therapy (CHART) trial [(6)] intensified local treatment by accelerating the RT course. When compared to conventional fractionation, there was a 9% absolute improvement in 2-year survival (29% v 20%; p=0.004) which provides evidence that intensification of local treatment only results in increased overall survival (OS). This result is supported by meta-analysis[(7)] reporting an absolute benefit for hyper-fractionated or accelerated radiotherapy over conventional fractionations of around 3% at 5 years.

Further evidence supporting intensification of local treatment comes from trials showing a survival benefit of concurrent chemo-radiotherapy over sequential[(5)]. This was achieved by chemotherapy increasing local tumour cell-kill and improving local control while maintaining the systemic effect of chemotherapy.

RT Dose Escalation

Increasing the radiotherapy dose has been associated with seemingly improved local control and OS in early-phase studies. However, RTOG 0617[(8)] comparing 60Gy in 30# to 74Gy in 37# once-daily showed that local control and OS were poorer on the higher dose. Reasons for this are being debated but it is clear that increasing RT by 2Gy per fraction per day with an increase in overall treatment time will not improve outcomes.

Isotoxic Radiotherapy

The MAASTRO group pioneered the concept of 'isotoxic RT' allowing for individualized dose escalation using hyperfractionated accelerated RT based on predefined and Mean Lung Dose (MLD) in stage I–III patients. This showed with 3D-CRT delivered two times a day over 4 weeks that increasing the radiation dose to pre-

For peer review only - http://bmjopen.bmj.com/site/about/guidelines.xhtml

specified normal tissue dose constraints could lead to increased tumour-control probability with the same normal tissue complication probability[9]. Survival of these stage III patients, all of whom were treated sequentially, were comparable to results expected from concurrent chemo-radiotherapy with acceptable acute and late toxicity. Since this study reported, Intensity modulated radiotherapy (IMRT) techniques have become routine practice improving targeting of radiation doses while decreasing doses to lungs and other normal tissue. This gives the potential to use the isotoxic approach to escalate the dose delivered to the target increasing tumour control probability with the same normal tissue complication probability[10].

Dose Acceleration and Escalation

Techniques that avoid prolongation of overall treatment time are attractive as they reduce the impact of accelerated tumour clonogen proliferation, which becomes clinically relevant for NSCLC approximately 3-4 weeks after radiotherapy initiation. A meta-analysis[7] of altered fractionations(10 trials, 2,000 NSCLC patients) showed modified fractionation improved OS as compared with conventional schedules(hazard ratio[HR]=0.88, 95% CI [0.80-0.97]; p=0.009), resulting in an absolute benefit of 2.5% at 5 years. CHART(54Gy in 36# over 12 days) remains one of the more commonly used schedules for radical treatment of NSCLC in the UK along with daily fractionated schedules delivering 55Gy in 20# over 4 weeks or 60-66Gy in 30-33# over 6-6½ weeks[11]. In current UK practice both CHART and the 4-week schedule are deliverable, with 99% of patients completing treatment, with survival outcome matching that seen in the original CHART study and very low levels of grade III/IV toxicity[12]. However, survival rates for all three regimens are low, and could be improved by dose-intensification.

Radiobiological modeling suggests that dose-escalation to improve the Tumour Control Probability (TCP) is likely to be more effective if the overall treatment time is fixed rather than fixing the dose per fraction, and dose-escalation typically adds 1-2% local control for each 1% increase in dose[13]. Therefore, CRUK funded 4 parallel dose-escalation studies to intensify accelerated schedules used in the UK. The first, CHART-ED (ISRCTN 45918260)[1] reported no dose limiting toxicities with dose-escalation(using additional twice-daily fractions of 1.8Gy) to 64.8 Gy in 17 days. This dose increase has the potential to increase local tumour control by 30%. The IDEAL CRT trial(ISRCTN 12155469) evaluated dose-escalation up to 73Gy in 30# over 6 weeks schedule with dose-escalation calculated on an individual patient basis according to either lung or oesophageal radiation dose. The study has established 68Gy to 1cc oesophagus as the MTD to be taken forward into future trials[2]. Both CHART-ED and IDEAL CRT report encouraging 2-year survival figures: 52% and 65% respectively.

Following the confirmation of low toxicity with the original IDEAL CRT trial, a further accelerated schedule was investigated where overall treatment time was shortened from 6 to 5 weeks by treating with RT twice a day on one day/week (not on chemotherapy day). The MTD to 1cc esophagus was reduced to 65Gy and the maximum prescription dose allowed was reduced to 71Gy in 30# (over 5 weeks). This portion of the study recruited 36 patients with a minimum 6-month follow up and no excess toxicity. This schedule will be taken forward and given sequentially in ADSCaN.

For peer review only - http://bmjopen.bmj.com/site/about/guidelines.xhtml

The two remaining studies, I-START (ISRCTN 74841904)[3] which individualized the RT dose similarly to IDEAL CRT using a 20# schedule following sequential chemotherapy and Isotoxic IMRT(NCT01836692)[4], where patients were treated twice-daily over 4 weeks to a maximum of 79.2Gy using pre-specified normal tissue doses have completed recruitment and neither report excess toxicity (personal communication).

The gold standard treatment for stage III NSCLC is concurrent chemo-radiotherapy [4]. However, in practice guidelines can only loosely define eligibility for the concurrent approach [14] and for many patients the concerns around fitness and the toxicity of this approach lead to chemo-radiotherapy being given sequentially [15]. A recent analysis of the National Cancer Database [16] suggests there is a basis for these concerns and that there are categories of patients for whom the higher toxicity levels associated with the concurrent approach could prove detrimental. At this point in time those categories are ill defined and novel strategies that integrate RT technological advances and radiobiological knowledge need evaluating in patients treated with sequential chemo-radiotherapy. This study aims to provide a framework for further development by identifying which is the best accelerated schedule to take forward into a randomised phase III study against conventionally fractionated radiotherapy.

METHODS AND ANALYSIS

ADSCaN is a non-blinded multicentre phase II study sponsored by NHS Greater Glasgow and Clyde Health Board and co-ordinated by the Cancer Research UK Clinical Trials Unit(CRUK CTU) based at the University of Glasgow. It is registered on the ISRCTN clinical trials database(ISRCTN47674500) and funded by Cancer Research UK's Clinical Trials Awards and Advisory Committee(CTAAC, Funder Reference: C9759/A16604). Radiotherapy quality assurance(QA) is supported by the National Radiotherapy Trial Quality Assurance(RTTQA) group. The study is included in the National Institute for Health Research(NIHR) Clinical Research Network portfolio(ID 35624) and is being conducted in accordance with the Declaration of Helsinki and Good Clinical Practice(GCP).

We hypothesize that sequential chemo-radiotherapy using accelerated, dose-escalated radiotherapy will improve progression-free survival(PFS), OS, time to local-regional failure, toxicity and cost-effectiveness compared to conventionally fractionated sequential chemo-radiotherapy treatment. ADSCaN will take the 4 dose escalated accelerated sequential chemo-radiotherapy schedules into a randomized phase II comparison with a UK standard sequential chemo-radiotherapy using state-of-the art radiotherapy.

Patient and Public Involvement Patient and carer representatives have been involved from the early stages of development of the ADSCaN study. These representatives had personal experience of sequential chemo-radiotherapy treatment and ensured the study reflected their priorities in developing the most appropriate chemo-radiotherapy regimens for this cohort of patients. Their engagement has been maintained through the National Cancer Research Institute(NCRI) Lung Clinical Studies Group(CSG) and the West of Scotland Cancer Research Network Consumer Research Panel. In particular, we thank Tom Haswell who has

For peer review only - http://bmjopen.bmj.com/site/about/guidelines.xhtml

become our consumer representative on the Trial Management Group to help oversee recruitment and conduct of the study.

Detailed results of the study will be given the study participants / their relatives on request and disseminated on the publically accessible websites of our funders, CR-UK, and other supportive charities (eg Roy Castle Lung Cancer Foundation.

Setting

ADSCaN opened on 22 August 2017 and will recruit 360 patients with a histological/cytological proven NSCLC across 40 UK radiotherapy centres, a list of which can be found on the NIHR Clinical Research Network Portfolio. Patients will be stage III, PS 0–1 and unsuitable for concurrent chemo-radiotherapy. Individual centres will randomise patients between standard radiotherapy (55 Gy in 20#) and one or more experimental arms. All sites will offer the standard arm, but may choose which experimental arms they wish to use (Figure 1: Flow diagram).

Participant screening and selection

Patients deemed inoperable by the lung multidisciplinary team (MDT) and suitable for sequential chemo-radiotherapy are invited to participate and provided with a patient information sheet (PIS, online supplementary appendix 1). Mandatory pre-randomisation investigations include: lung function tests, contrast CT scan of thorax/upper abdomen within 4 weeks prior to randomisation and fluorodeoxyglucose positron emission tomography (FDG-PET) with CT (or MRI) brain scan if patients have not these investigations prior to starting induction chemotherapy.

Inclusion Criteria

- Histologically/cytologically confirmed stage III NSCLC
- ECOG Performance status (PS) 0-2 (PS 2 only included if the local investigator deems general condition is explained by disease or the primary chemotherapy treatment).
- Inoperable disease
- Inoperable disease, unsuitable for concurrent chemo-radiation, in the opinion of the treating Oncologist
- Patients with complete response, partial response or stable disease on CT assessment after 2 cycles of platinum-based chemotherapy
- Aged ≥16
- Adequate PFT results: FEV1 and/or KCO≥40% of predicted.

Exclusion Criteria

- Previous/current malignant disease likely to interfere with the protocol treatment or comparisons
- Clinically significant interstitial lung disease

Informed consent

For peer review only - http://bmjopen.bmj.com/site/about/guidelines.xhtml

Eligibility is confirmed by a clinician and patients are given ≥24 hours to consider the PIS and ask questions prior to written informed consent being taken.

Randomisation

Randomisation occurs after completion of chemotherapy. Once a patient is deemed eligible for the study and has consented to participate, the CRUK CTU, Glasgow, is contacted to confirm eligibility, and subsequently the patient is randomised to one of the available ADSCaN arms offered on-site and issued with a participant identification number. Patient enrolment details are confirmed by email.

Minimisation incorporating a random factor is used to allocate patients between treatment arms A:B:C:D:E so that an overall study ratio of 2:1:1:1:1 is achieved. The following stratification factors are used:

- Hospital
- Performance Status
- Intention to use IMRT
- Histological type
- Stage
- EGFR mutation status
- Gross Tumour Volume(GTV)
- Haemoglobin
- Serum albumin
- Sex
- Weight loss since diagnosis

While on study, participants will not co-enrol in other clinical trials offering therapeutic intervention.

Standard care

In patients with inoperable stage III NSCLC who are unsuitable for concurrent chemo-radiotherapy, sequential chemo-radiotherapy is the standard of care.

Patients who are eligible for this trial will receive 2-4 cycles of platinum doublet neo-adjuvant chemotherapy. The recommended non-platinum agents are pemetrexed or vinorelbine for non-squamous carcinoma and gemcitabine or vinorelbine for squamous carcinoma. The TMG / DMEC are aware that the standard of care for the systemic treatment of stage III NSCLC may evolve over the course of this study and will consider the evolving evidence on newer approaches such as immunotherapy. **RT intervention and planning**

4D-CT with intravenous contrast should be used for all ADSCaN patients to account for tumour motion during breathing. The whole thorax(cricoid to L2) should be covered to allow dose volume histograms to be calculated for the lung, heart, spinal cord, brachial plexus, great vessels, proximal bronchial tree and the oesophagus.

The input of a thoracic radiologist is recommended when defining the identifiable tumour and involved lymph nodes to give the GTV. The thoracic radiologist should work with a qualified radiation oncologist specialised in thoracic malignancies to produce a motion adapted GTV.

For peer review only - http://bmjopen.bmj.com/site/about/guidelines.xhtml

The 4D-GTV is expanded by 5mm to create the Clinical Target Volume (CTV). The CTV will be expanded to include microscopic spread and may be edited to account for anatomical barriers but only if it is not thought to invade the structure.

CTV to planning target volume(PTV) expansion takes into account patient set-up uncertainties.

- If a centre is using per fraction Cone Beam CT(CBCT) imaging and online correction for treatment verification, the CTV should be expanded by 5mm isotropically to create the PTV.
- If a centre is not CBCT imaging per fraction, the CTV should be expanded by 7mm axially and 9mm cranio/caudally to create the PTV.

Prescribed dose and fractionation

Radiotherapy schedules should begin on a Monday where possible to reduce overall treatment time. Schedules are summarised in Table 1 and dose constraints for organs at risk in Table 2.

All patients recruited at a centre must be treated with the same planning technique irrespective of randomisation arm. The use of IMRT is strongly recommended(and mandated for Arm E) using 5 or more fields in an IMRT plan or an arc technique may be used.

Follow-up

Patients are followed up monthly for 3 months, then 3-monthly to 2 years, 6-monthly to 3 years then annually. A late toxicity assessment will be performed at each visit.

Withdrawal

Patients can withdraw from the trial at any time without any effect on clinical care.

Quality of Life

Quality of life(QoL) as measured by EQ-5D will be completed at all study visits and weekly during treatment.

Data Collection

All information collected during the course of the study will be kept strictly confidential. Data will be entered on to an electronic case report form system with built-in edit checks to ensure high quality data. Sites will be monitored centrally by checking incoming data for compliance with the protocol, data consistency, missing data and timing.

Statistical considerations

The comparison of each experimental arm to standard treatment has 80% power to detect a 33% increase in median PFS(from 8 to 10.7 months) at the 20% 1-sided level of statistical significance [17]. A 33% improvement in PFS was selected as it was felt realistic and that an improvement of this magnitude was required to ensure UK support for any subsequent phase III. There is no formal calculation of sample size for the “pick-the-

For peer review only - http://bmjopen.bmj.com/site/about/guidelines.xhtml

winner" element; if more than one experimental arm passes the screening threshold a choice will be made using efficacy, toxicity, QoL and health economics (HE) evidence.

Primary Analysis

The primary statistical analysis will be based around a Cox model fitted to PFS incorporating terms for study arm and other prognostic factors that reflect case-mix. Model fit and the proportional hazards assumption will be checked by plotting Schoenfeld residuals and a test for goodness of fit[(18)]. The assessment of the effect of an experimental arm relative to standard and experimental arms relative to one another will be made via the hazard ratios(and associated p-values) derived from this model.

Secondary Analysis

The analysis of OS and time to local-regional failure will be analysed as for PFS.

Toxicity will be assessed using NCI CTCAE v4.03. Late effects will be monitored. The worst grade will summarized and compared between study arms using the Mann-Whitney U-test. HE analysis will be undertaken from the perspective of the UK National Health Service(NHS), adhering to the National Institute for Health Care Excellence(NICE) reference case to assess cost-effectiveness of the experimental arms compared to current standard. QoL will be assessed using EQ-5D and parametric extrapolation undertaken on the PFS and OS curves to generate lifetime outcomes and compare across study arms in terms of quality adjusted life year(QALY) gains.

Interim Analysis

Study data will be reviewed at least annually by an Independent Data Monitoring Committee (IDMC). Safety stopping in the experimental arms will be monitored continuously based on grade 4/5 acute radiation toxicity.

For peer review only

Changes to the protocol after the start of the trial

This document is consistent with ADSCaN study protocol (Version 1.2, dated 20th January 2017). There have been no significant changes to the protocol since the start of the study.

End of the trial

The study will close 12 months after the last patient has recruited or when 305 PFS events have been observed (whichever is later). The chief investigator and/or the trial management group (TMG) have the right at any time to terminate the study for clinical or administrative reasons. The end of the study will be reported to the REC and Regulatory Authority (where applicable) within the required timeframes.

Quality Assurance programme

The trial is subject to a RT QA programme, which includes pre-trial and on-trial components. Dosimetry audit will be performed in centres where a recent relevant external audit has not been completed. Every attempt will be made to streamline with previous national and international audits. The QA programme for the study is

For peer review only - http://bmjopen.bmj.com/site/about/guidelines.xhtml

coordinated by the UK RTTQA Group. The details of the programme can be found at the RTTQA website, http://www.rttrialsqa.org.uk.

The pre-trial QA includes:

- Facility questionnaire.
- Outlining benchmark case. Target volumes and organs at risk(OAR) are delineated on a CT data set provided. The OAR should be outlined according to the ADSCaN OAR Atlas.
- Planning benchmark case. Planned according to the trial protocol on a pre-outlined CT(GTV, CTV, PTV and OAR).

Centres that have previously completed pre-trial QA programmes for the individual trial arms, CHART-ED, IDEAL, I-START and Isotoxic IMRT, may be eligible for QA streamlining.

The on-trial QA includes Individual Case Reviews (ICR) to monitor protocol adherence:

- Prospective ICR for at least the first patient from each centre for each trial arm. Retrospective ICR for selected cases.

ETHICS AND DISSEMINATION

Adverse events are collected (according to the CTCAE V.4.0 grading system) at each trial visit and all adverse events on study are reported to the CRUK CTU Glasgow and followed until they resolve or stabilise. Acute and late radiation toxicities continue to be recorded at each follow-up visit.

Trial monitoring and oversight

Formal on site data monitoring activities are performed as part of the ADSCaN study. Each site will receive telephone and on-site monitoring visits during the course of the study.

Data is reviewed by an IDMC (comprising two clinicians and one statistician) and Trials Steering Committee. The TMG coordinates and manages the trial's day-to-day activities and is comprised of health professionals, a patient representative and members of the direct study team, including the principal investigators for each of the experimental arms.

Dissemination

Data from all centres will be analysed together and published promptly. Individual participants may not publish data concerning their patients that are directly relevant to questions posed by the trial until the TMG has published its report. The TMG will form the basis of the Writing Committee and advise on publications. The trial will be publicised at regional and national conferences. The final results will be presented at scientific meetings and published in a peer-reviewed journal(authorship will be according to the journal's guidelines). In addition a lay summary of the results will be produced for interested parties for example, Cancer Research UK. The CTU is committed to furthering research by sharing de-identified individual-patient data(IPD) from its studies with others in the field who wish to use the data for high quality science and we are happy to consider proposals from researchers and will share IPD to the maximum extent.

For peer review only - http://bmjopen.bmj.com/site/about/guidelines.xhtml

For peer review only

Figure 1

ADSCaN Trial Schema

For peer review only

For peer review only - http://bmjopen.bmj.com/site/about/guidelines.xhtml

Trial status

The study opened to recruitment on 22nd August 2017.

Author contributions

MH conceived of the study. MH, CF-F, DL, JL, JF, JP, EM, KB initiated the study design and CL, RS and AS helped with implementation. MH is the grant holder. JP provided statistical expertise in clinical trial design. All the authors contributed to refinement of the study protocol and approved the final manuscript. TH is the patient representative inputting into study design and Implementation.

Acknowledgements The authors would like to acknowledge the support of the study sponsor NHS Greater Glasgow & Clyde, study funder CRUK and the patients who consent to enter the study. In addition we would like to acknowledge the individual contributions of Joe Maguire, The Clatterbridge Cancer Centre, Wirral, Emma Parsons, Elizabeth Miles(National Radiotherapy Trials Quality Assurance(RTTQA) Group, Mount Vernon Hospital, Middlesex, UK), Joanne McGarry(NHS Greater Glasgow & lyde), Stephen Clark, Lindsey Connery, Calum Innes(CRUK Clinical Trials Unit, Institute of Cancer Sciences, University of Glasgow, UK).

Contributors All the authors contributed to refinement of the study protocol and approved the final manuscript.

Funding This research is funded by Cancer Research UK's(CRUK) Clinical Trials Awards and Advisory Committee(CTAAC). Grant reference number A16604.

Competing interests None declared.

Ethics approval West of Scotland Research Ethics Committee, West of Scotland REC 1, 8th November 2016 (REC reference: 16/WS/0165)

Sponsor: NHS Greater Glasgow and Clyde (Greater Glasgow Health Board: J B Russell House, Gartnavel Royal Hospital, 1055 Great Western Road, Glasgow, G12 0XH)

The Sponsor is responsible for confirming there are proper arrangements for the initiation and management of the study. The majority of the Sponsor's responsibilities have been delegated to the Chief Investigator(CI) who performs these via the CRUK CTU as the co-ordinating centre for the trial. As such, the main role of the Sponsor is to ensure that the CI and CRUK CTU fulfil their responsibilities as outlined in the Clinical Trial Agreement and to ensure that any identified "risks" either have controls or action points put in place.

For peer review only

For peer review only - http://bmjopen.bmj.com/site/about/guidelines.xhtml

For peer review only

For peer review only - http://bmjopen.bmj.com/site/about/guidelines.xhtml

Table 1: Summary of Trial Arms

Trial arm	Total dose	Total fractions	fractions per day	Total number of days
**Arm A:** Standard Arm	55.0Gy	20	1	26-28
**Arm B:** CHART-ED	54.0Gy<br>10.8Gy	36<br>6	3<br>2	Days 1-12<br>Days 15-17
**Arm C:** IDEAL	63-71Gy	30	1 with 1 B.D treatment per week	33-35
**Arm D:** I-START	55-65Gy	20	1	26-28
**Arm E:** Isotoxic IMRT	61.2-79.2Gy	34-44	2	23-30 depending on dose-escalation

For peer review only

For peer review only - http://bmjopen.bmj.com/site/about/guidelines.xhtml

Table 2: Dose constraints for all trial arms

Volume	Arm A: Standard	Arm B: CHART-ED	Arm C: IDEAL-CRT	Arm D: I-START	Arm E: Isotoxic IMRT
CTV	V95% >99%	V95% > 99%	V95% > 99%	V95% > 99%	Optimal: CTV $D_{(Total\ Vol\ -1cc)}$ ≥95%<br>Mandatory: $CTV^{MeanDose}$+/- 1% of prescribed dose
PTV	V95% > 90%<br>V90% >98%	V95% > 90%<br>V90% > 98%	V95% > 90%<br>V90% > 98%	V95% > 90%<br>V90% > 98%	Optimal: PTV $D_{(Total\ Vol\ -1cc)}$ ≥90%<br>PTV D95% ≥95%<br>Mandatory: PTV $D_{(Total\ Vol-1cc)}$ ≥85%<br>PTV D95% ≥90%<br>PTV D1cc ≤107%
Spinal Canal	D0.1cc <47Gy	D0.1cc <46Gy	D0.1cc < 48Gy	D0.1cc < 47Gy	D1.0cc ≤ EQD2 50Gy
Lung-GTV	V20Gy <35%	V20Gy <35%	V20Gy < 35%		Mean Dose ≤20Gy(physical dose)
Brachial Plexus	D0.1cc <55Gy	D0.1cc <64.3Gy	D0.1cc < 65Gy<br>D30% < 60Gy(Ipsilateral BPlexus)	D0.1cc <55Gy	D1.0cc ≤ EQD2 66Gy
Heart	D100% < 36Gy<br>D67% < 44Gy<br>D33% < 57Gy	D100% < 48Gy<br>D67% <57Gy	D100% < 44Gy<br>D67% < 52Gy<br>D33% < 59Gy	D100% < 36Gy<br>D67% < 44Gy<br>D33% < 57Gy	D1.0cc ≤ EQD2 76Gy<br>Mean Dose ≤46Gy(physical dose)
Oesophagus	D0.1cc <105%	D0.1cc <105%	D1.0cc < 65y		
Lung Contra	V5Gy <60%*		V5Gy <60% *	V5Gy < 60%*	
Mediastinal Env					D1.0cc ≤ EQD2 76Gy

* Optimal constraint (i.e. not mandatory limit)

For peer review only - http://bmjopen.bmj.com/site/about/guidelines.xhtml

REFERENCES

1. Aupérin A, Le Péchoux C, Rolland E, Curran W, Furuse K, Fourne P et.al. : Meta-analysis of concomitant versus sequential radio-chemotherapy in locally advanced non-small-cell lung cancer. J Clin Oncol 2010;28(13):2181-90
2. Hatton M, Hill R, Wilson P, Atherton P, Morgan S, Dickson J, Murray K, Paul J: Continuous Hyperfractionated Accelerated RadioTherapy – Escalated Dose(CHART-ED): A Phase I study: Radiotherapy Oncology. 2016;118:471-77. ISRCTN: 45918260
3. Landau DB, Khan I, Baker A et.al. IDEAL-CRT: A Phase 1/2 Trial of Isotoxic Dose-Escalated Radiation Therapy and Concurrent Chemotherapy in Patients with Stage II/III Non-Small Cell Lung Cancer. Int J Radiat Oncol Biol Phys 2016; 95:1367-1377 ISRCTN: 12155469
4. I-START Study ISRCTN: 74841904
5. Haslett K, Franks K, Hanna GG, Harden S, Hatton M, Harrow S et.al. Protocol for the isotoxic intensity modulated radiotherapy(IMRT) in stage III non-small cell lung cancer(NSCLC): a feasibility study. BMJ Open 2016;6:e010457.doi:10.1136/bmjopen-2015-010457
6. Saunders M, Dische S, Barrett A et al., for the CHART Steering Committee. Continuous hyperfractionated accelerated radio-therapy(CHART) versus conventional radiotherapy in non-small-cell lung cancer: A randomised multicentre trial. Lancet 1997; 350:161–165.
7. Mauguen A, Le Péchoux C, Saunders MI, Schild SE, Turrisi, Baumann M, et.al. Hyperfractionated or Accelerated Radiotherapy in Lung Cancer: An Individual Patient Data Meta-Analysis. J Clin Oncol. 2010; 28:2181-90
8. Bradley JD, Paulus R, Komaki R et al. Standard-dose versus high-dose conformal radiotherapy with concurrent and consolidation carboplatin plus paclitaxel with or without cetuximab for patients with stage IIIA or IIIB non-small-cell lung cancer(RTOG 0617): a randomised, two-by-two factorial phase 3 study. Lancet Oncol 2015; 16(2):187-99
9. van Baardwijk A, Wanders S, Boersma L, Borger J, Oellers M, Dingemans AMC, et.al. Mature results of an individualized radiation dose prescription study based on normal tissue constraints in stages I to III non-small-cell lung cancer. J Clin Oncol 2010; 28(8):1380-6
10. Chan C, Lang S, Rowbottom C, Guckenberger M, Faivre-Finn C; IASLC Advanced Radiation Technology Committee. Intensity-modulated radiotherapy for lung cancer: current status and future developments. J Thorac Oncol. 2014;9(11):1598-608.
11. Helbrow J, Bayman N, MacNicoll F, Faivre-Finn C: Concurrent chemo-radiotherapy(cCTRT) for locally advanced unresectable non-small cell lung cancer(LA-NSCLC): A national survey of current practice. Lung Cancer 2012; 75(1):S50-S51
12. Pemberton LS, Din OS, Fisher PM, Hatton MQ. Accelerated radical radiotherapy for non-small cell lung cancer(NSCLC) using two common regimens: a single centre audit of outcome. Clinical Oncology 2009;21:161-7
13. Fenwick JD, Nahum AE, Malik MI et al. Escalation and Intensification of Radiotherapy for Stage III Non-small Cell Lung Cancer: Opportunities for Treatment Improvement. Clinical Oncology 21:343-360;2009
14. Lim E, Baldwin D, Beckles M et.al. Guidelines on the radical management of patients with lung cancer. Thorax 2010:65:Suppl III.
15. De Ruysscher D, Botterweck A, Dirx M et al. Eligibility for concurrent chemotherapy and radiotherapy of locally advanced lung cancer patients: a prospective, population-based study. Ann Oncol 2009;(1):98-102
16. Miller ED, Fisher JL, Hagland KE, et al. The addition of chemotherapy to radiation therapy improves survival in elderly patients with stage III NSCLC. JTO 2018;13:426-435
17. Rubinstein LV, Korn EL, Freidlin B, et.al. Design Issues of Randomized Phase II Trials and a Proposal for Phase II Screening Trials Journal of Clinical Oncology 2005 23:28, 7199-7206

For peer review only - http://bmjopen.bmj.com/site/about/guidelines.xhtml

18. Parzen, M; Lipsitz, SR: A global goodness-of-fit statistic for Cox regression models BIOMETRICS Volume: 55 Issue: 2 Pages: 580-584: JUN 1999

For peer review only

For peer review only - http://bmjopen.bmj.com/site/about/guidelines.xhtml

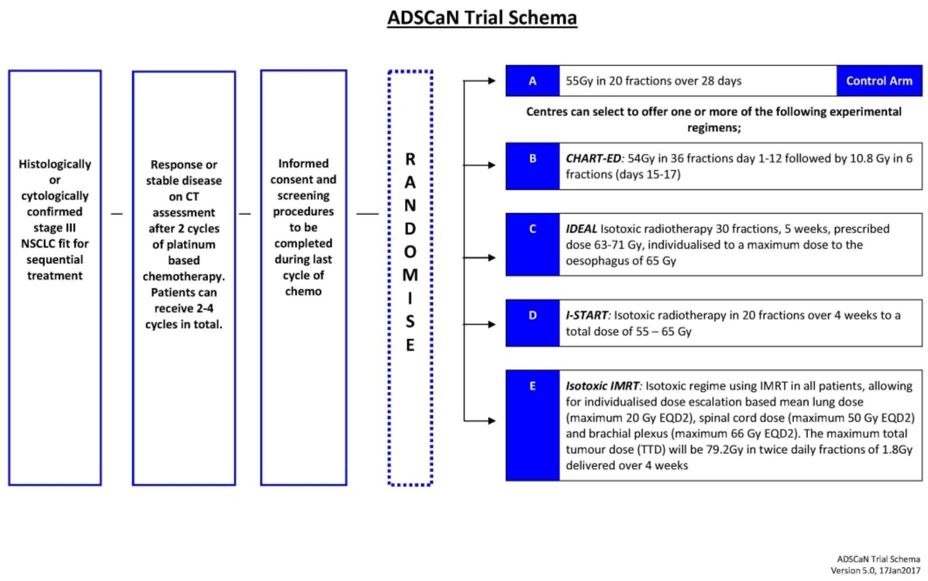

Figure 1 ADSCaN Trial Schema

297x209mm (300 x 300 DPI)

(Form to be on hospital headed paper)

INFORMATION SHEET FOR PATIENTS/ VOLUNTEERS IN CLINICAL RESEARCH PROJECT

Title of Project:
ADSCaN - A Randomised Phase II trial of **A**ccelerated, **D**ose escalated, **S**equential **C**hemo-r**a**diotherapy in **N**on-small cell lung cancer

Invitation Paragraph
We would like to invite you to take part in a research study (also called a clinical trial) because your doctors are considering a course of radiotherapy for your lung cancer. Before you decide whether or not to take part, it is important for you to understand why the research is being done and what it will involve for you. Please take time to read the following information carefully and discuss it with friends, family or your General Practitioner (GP) if you wish. Ask us if there is anything that is not clear or if you would like more information. Take time to decide whether or not you wish to take part.

Summary
When we refer to 'your doctor' in this information sheet we are referring to the consultant oncologist responsible for your care relating to your lung cancer in hospital.

Your doctor will have explained that the combination of chemotherapy followed by radiotherapy is the best treatment for your cancer.

You may already have started chemotherapy. Radiotherapy treats the cancer by using high-energy x-rays to destroy cancer cells. This treatment is given in hospital radiotherapy departments in short sessions that only take a few minutes, each session is known as a fraction. The fractions add up to a full course of treatment.

It is thought that giving a higher dose of radiotherapy during the course of treatment will destroy more of the cancer cells, but this has to be done with no more measurable damage to the nearby healthy cells.

Part 1 tells you the purpose of this trial and what will happen to you if you take part.

Part 2 gives you more detailed information about the conduct of the trial.

PART 1

What is the purpose of this trial?
You have been diagnosed with a certain form of lung cancer, called non-small cell lung cancer (NSCLC). Patients with locally advanced lung cancer are often treated with chemotherapy and high dose radiotherapy when surgery is not an option. This trial is looking at different courses of radiotherapy treatment for patients with non-small cell lung cancer. In order to try to improve the result of treatment a number of UK trial

For peer review only - http://bmjopen.bmj.com/site/about/guidelines.xhtml

groups have been developing higher dose radiotherapy courses and testing their safety. Four courses that increase the radiotherapy dose delivered to the tumour have been developed and trials have now shown us the right dose for these courses. We now wish to test these different radiotherapy courses against each other to see which is most promising and compare them to the current UK standard course of treatment.

In some of these new courses of treatment, the fractions are given once a day and in others there are two or three fractions per day. This new study will try to identify which course of treatment is the most promising and should be tested further in a future trial; the ultimate aim is to increase the cure rate for patients with inoperable lung cancer. Your hospital is able to offer one or more of these new treatment courses.

Why have I been invited?

You have been invited to take part in this trial because you have non-small cell lung cancer which can be treated with radiotherapy after a course of chemotherapy.

Do I have to take part?

Taking part in the trial is entirely voluntary. It is up to you to decide whether or not to take part. If you do decide to take part, you will be given this information sheet to keep and asked to sign a consent form.

If you decide to take part you are still free to withdraw at any time and without giving a reason. If you do not wish to participate this will not affect the standard of your treatment and you will be offered whatever treatment your consultant thinks is best for you.

How are treatment options decided?

When a treatment is discovered we don't always know which way of treating patients is best. To find out, we need to make comparisons between the different treatments. To do this we put patients randomly into groups and give each group a different treatment; the results are compared to see if one treatment is better than the others.

To try to make sure the groups are the same to start with, each patient is put into a group by chance. This process is called randomisation and, if you agree to take part, you will be put into a group by chance when neither you, nor your doctor can pick the treatment group. You will receive one of the following ticked radiotherapy treatment course *(the treatment courses available at this hospital are ticked)*:

<< TO BE POPULATED AS PER THE ARMS SITE IS PARTICIPATING IN >>

- ☑Standard Radiotherapy
- ☐CHART-ED
- ☐IDEAL
- ☐I-START
- ☐ISOTOXIC IMRT

In this trial we are aiming to randomise 120 patients to standard radiotherapy and 60 patients to each of the four new courses of radiotherapy: CHART-ED, IDEAL, I-START, ISOTOXIC IMRT.

For peer review only - http://bmjopen.bmj.com/site/about/guidelines.xhtml

What will happen to me if I take part?

Standard radiotherapy is given over 4 weeks with 20 fractions given on Monday to Friday. If you agree to take part in the study, the length of the course of radiotherapy will vary so your course may be slightly shorter or longer than the standard lasting between 17 days and 5 weeks in total.

Before starting radiotherapy treatment, we will check your general health and weight, which includes taking a history of your previous health and current medications and carry out a physical examination. You will have all of the normal tests that are performed before radiotherapy; these include a CT scan of your chest and abdomen, chest x-ray, blood tests, breathing tests (Pulmonary Function Tests) and a heart trace (ECG). You will also have a CT or MRI scan of your head that may be an additional test done for the trial. These tests are done to make sure you are suitable for the trial.

You will then attend the Radiotherapy Department for a planning-CT scan. The doctors, radiographers and physicists will use this CT scan to plan your treatment. Once the radiotherapy has been planned, you will attend the Radiotherapy Department for a "planning" session. If this is satisfactory, your radiotherapy treatment will start a few days later.

If any results of the tests carried out mean you cannot participate in the trial, you will be unable to enter the trial and your treating doctor will ensure that you will receive the standard treatment that is best for you.

At some visits to hospital your doctor or nurse will ask you some extra questions about how you are feeling and if you have noticed any side effects from your radiotherapy. After any radiotherapy treatment, you will continue to be seen by your doctor to check for any side effects and see how well your treatment has worked. You will see your doctor in clinic as you would normally, but you may be asked some additional questions at these visits.

You will receive a course of radiotherapy treatment outlined in the table:

For peer review only - http://bmjopen.bmj.com/site/about/guidelines.xhtml

Course of Radiotherapy	Description	Sessions per day	Number of Weeks
**Standard Radiotherapy**	One radiotherapy session per day on weekdays over 4 weeks.	1	4
**CHART-ED**	Three radiotherapy sessions per day over 12 days (including weekend) without a gap. This is followed after a weekend gap of 2 days by two radiotherapy sessions per day over 3 days.<br><br>Treatment will last 17 days in total. It will start on a Monday and you will stay in hospital whilst you receive treatment. You will have a break and be allowed to go home for the second weekend (get home on the Friday afternoon and return on the Monday morning).	3	2.5
**IDEAL**	One-to-two radiotherapy sessions per day on weekdays over 5 weeks.	1	5
**I-START**	One radiotherapy session per day on weekdays over 4 weeks.	1	4
**ISOTOXIC IMRT**	Two radiotherapy sessions per day on weekdays over 4 weeks.<br><br>You will be treated with indivdualised (isotoxic and targeted (IMRT) doses of radiotherapy. The radiotherapy session will take around 15 minutes each time and there will be a break of at least 6 hours between the two sessions given each day.<br><br>If you do not live close enough to the hospital to return home during the break between radiotherapy sessions, you may be able to stay in hospital.	2	4

You will have blood tests whilst you are receiving the radiotherapy, which would happen as part of your standard care. 1-2 teaspoons or 5-10ml of blood will be needed.

You will have your breathing tests repeated at three months and one year after you have finished your radiotherapy.

For peer review only - http://bmjopen.bmj.com/site/about/guidelines.xhtml

You will continue to have chest x-rays and CT scans regularly to make sure your lung cancer has not come back. The chest x-rays would all be done as part of your standard care. You will have a CT scan at 3, 6, 12 18, 24 and 36 months after radiotherapy. You would have the CT scans at 3 and 12 months after radiotherapy as part of your standard care, however in this trial you will have the additional scans so that we can check your response to radiotherapy more closely.

In addition, we will ask you to fill out questionnaires called "Quality of Life" questionnaires before the beginning of the trial, during radiotherapy treatment and at every clinic visit after treatment. These questionnaires ask you about how you feel and take about 10 minutes to complete. Some of these questions are personal and you can refuse to answer them if you wish. The information you provide is for research purposes and will remain strictly private and confidential.

What is the standard treatment?

The standard radiotherapy treatment most commonly used in the UK is 20 fractions given over 28 days. This is a lower dose than those of the experimental arms being tested in this trial.

What are the possible benefits of taking part?

The information we get from this trial may help us to decide if some radiotherapy treatments are better than others are and may help us to treat future patients with the same disease better.

What are the possible disadvantages and risks of taking part?

There are side effects associated with the radiotherapy, which are detailed below. If you are randomised to receive experimental radiotherapy these doses are slightly higher than the standard dose, therefore side effects may be more likely to occur or may be more severe. You can stop the trial treatment at any time if you or your doctor feels that the side effects are a problem for you.

Information on side effects and the treatment of these side effects will be closely monitored by doctors involved in the trial. In addition, the trial will be carefully monitored by an independent group of specialists who will regularly advise all the investigators about the safety of the trial.

As part of this trial, you will be exposed to radiation from computed tomography (CT) scans and from your radiotherapy treatment. Your radiotherapy treatment uses strong (high energy) X-rays to treat your cancer, whereas CT scans use lower energy X-rays to form pictures of your body, which provide detailed information about your cancer. The dose of radiation you receive from a CT scan is much less than the dose of radiation you will receive from your radiotherapy.

The risk of radiation exposure associated with chest x-ray is extremely small and relates to a lifetime risk of induced cancer of 0.0005%, which is 5 in one million.

For peer review only - http://bmjopen.bmj.com/site/about/guidelines.xhtml

The total radiation dose due to all forms of imaging examinations carried out in the course of the study corresponds to an estimated lifetime risk of induced cancer of 0.3 – 1.2%. Of this total imaging dose, around two-thirds comes from examinations that would be carried out in the course of normal treatment anyway. The remaining non-routine dose corresponds to a lifetime induced cancer risk of 0.01 – 0.67%.

In a previous lung radiotherapy study that delivered escalated doses, cancers most probably induced directly by the radiation treatment were seen in 2 of 112 patients.

There are no radiation risks associated with MRI. However, some people are allergic to the contrast fluid that is injected as part of the CT or MRI scan procedure. Sometimes contrast fluid can cause damage to the kidneys. Both of these are rare reactions. If you have previously had a bad reaction to contrast fluid, please let your doctor know.

You may be given a higher dose of radiotherapy so the side effects may be more than if you had the standard dose. This is monitored extremely carefully to ensure the amount received by normal tissues is within safe limits and is also safe for you. You will need to tell your doctor or nurse about any side effects you experience.

What are the side effects of radiotherapy?

Early side effects

These can occur during the four weeks of radiotherapy or soon after the treatment has finished. They almost always settle within 2 months of finishing radiotherapy.

Soreness when swallowing (Oesophagitis) - this can start towards the middle of treatment and may be at its worst a week or two after treatment has finished. It should then gradually settle down. There are medicines available that may help this.

Cough and shortness of breath - the radiotherapy may cause inflammation in the lung which can cause shortness of breath and cough. There are medicines which may help control these symptoms.

Skin reaction – skin over the treated area may become red as the treatment progresses.

Tiredness – you will probably feel more tired than usual. This will wear off gradually, but may last for a number of weeks after treatment finishes.

Late side effects

These can occur several months after the radiotherapy has finished. They are usually permanent.

Soreness and difficulty swallowing - Unusually, radiotherapy can cause scarring and narrowing of the oesophagus. This can be helped by medicine but may need further investigation and treatment

Lung scarring (Fibrosis) – The radiotherapy will damage some of the normal lung tissue around the cancer which will, after six to nine months, cause some scarring. This can cause breathlessness, which in a minority of cases can be severe.

For peer review only - http://bmjopen.bmj.com/site/about/guidelines.xhtml

Skin colour change – Sometimes the skin over the treated area is left darker or lighter than it was before the radiotherapy. You will receive information about skin care when you start your treatment.

Effect of treatment on fertility and contraception

Radiotherapy can cause damage to the ovaries or testes. This may cause permanent infertility (inability to have a child) but the risk is very small when the lungs are being treated.

If it is possible you may conceive (i.e. you are of childbearing age), you will be asked to have a pregnancy test before starting radiotherapy treatment.

It is still possible for women of childbearing age to become pregnant, and men to father a child during treatment. Therefore, you or your partner should use effective barrier methods of contraception during treatment (e.g. condoms), and for six months after treatment, because of the risk to the unborn child. You would be advised to do this even if you were not taking part in this trial. In the event that you or your partner becomes pregnant while you are taking part in the trial you should consult your doctor immediately and inform your research nurse. Your research nurse will then report the pregnancy to the Cancer Research UK Clinical Trials Unit Glasgow, who are running the trial. Should you or your partner become pregnant during the trial you will be provided with additional information about this process.

What are the risks from radiation?

As part of this trial, you will be exposed to radiation from computed tomography (CT) scans and from your radiotherapy treatment. Your radiotherapy treatment uses strong (high energy) X-rays to treat your cancer. CT scans use lower energy X-rays to form pictures of your body which provide detailed information about your cancer. The dose of radiation you receive from a CT scan is much less than the dose of radiation you will receive from your radiotherapy.

As part of this trial, you will have more scans than with standard care. This additional radiation may slightly increase the risk of your developing cancer at a later date. However the risk is considered to be small when compared to the possible benefits of treatment.

In addition to these risks, some people are allergic to the contrast which is injected at the time of the CT scans. Sometimes contrast can cause damage to the kidneys. If you have previously had a reaction like this, please let your doctor know.

What happens when the research trial stops?

If the research trial stops and you require treatment you will receive the standard radiotherapy dose you would have been given if you had not agreed to take part in the trial.

For peer review only - http://bmjopen.bmj.com/site/about/guidelines.xhtml

If you have already received radiotherapy treatment you will continue to be followed up in the normal way.

What if there is a problem?
Any complaint about the way you have been dealt with during the trial or any possible harm you might suffer will be addressed. The detailed information concerning this is given in Part 2 of this information sheet. If you have any concerns or complaints you should contact your doctor in the first instance.

Will my taking part in the trial be kept confidential?
Yes. We will follow ethical and legal practices and all information about you will be handled in confidence. The details are included in Part 2.

This completes Part 1 of the information sheet.

If the information in Part 1 has interested you and you are considering taking part, please read the additional information in Part 2 before making any decision.

PART 2

What if I change my mind?
If you do decide to take part in this trial then you are still free to withdraw at any time without giving a reason. This will not affect your future care in any way but we would still like to know how you are, and with your permission, will request this information from your doctor. Please let your doctor know if you wish to withdraw and he/she will carry on your care in the normal way.
If you withdraw, you will be asked to clarify which part of the trial you are withdrawing from. You may withdraw from some parts or all parts. Depending on your response, we will know what information we can keep and whether we can still collect information on your progress.

What if new information becomes available?
Sometimes during the course of a research project, new information becomes available about the treatment that is being studied. If this happens, your doctor will tell you about it and discuss with you whether you want to continue in the trial. If you decide to withdraw, your doctor will make arrangements for your normal care to continue. If you decide to continue in the trial you will be asked to sign a new consent form.

Also, on receiving new information, your doctor might consider it to be in your best interest to withdraw you from the trial. He/she will explain the reasons and arrange for your usual care to continue.

What if something goes wrong?
If you have a concern about any aspect of this trial, you should ask to speak with the research doctor/nurse, who will do their best to answer your questions.

For peer review only - http://bmjopen.bmj.com/site/about/guidelines.xhtml

If taking part in this research trial harms you, there are no special compensation arrangements. If you are harmed due to someone's negligence, then you may have grounds for a legal action but you may have to pay your legal costs. Regardless of this, if you wish to complain or have any concerns about any aspect of the way you have been approached or treated during the course of this trial, the normal National Health Service complaints mechanism is available to you.

If you have private medical insurance, you may wish to check with your company before agreeing to take part in the trial to ensure that participation in the trial will not affect your insurance cover.

Additional Research – translational studies

We will be asking you whether we can collect some samples to allow us to carry out further research studies. These samples will include a sample of the original tumour taken at the time of your original biopsy, and will have been stored in the pathology department since then. We may also ask you for a blood test, which could be carried out at the same time as routine blood tests.

Samples from patients participating in clinical trials such as ADSCaN, are extremely valuable as accurate clinical information is collected on the same patients. This information, including whether the cancer responds to treatment or whether the disease comes back in the future, can be used together with the information discovered in the laboratory to help answer questions about lung cancer and its treatment.

These additional studies will not affect your treatment in any way. If you decide not to take part in the collection of samples for laboratory research, this will not affect your participation in the clinical part of ADSCaN, or your relationship with your doctor.

Any unused parts of the samples collected will be stored for as long as possible, in case further investigations are developed in the future, which may help us understand more about the response of your type of cancer to these therapies. The samples may also be used for other studies in the future relating to lung cancer, and may involve genetic tests to look at genetic influences on these cancer types. Any such studies will have to be approved by the ADSCaN Trial Management Group and external ethics committees. We cannot describe here all the potential tests which may be done on any samples stored and if you feel uncomfortable about this please do not participate.

Will my taking part in this trial be kept confidential?

You can be assured that any data collected during the course of this trial and any of the results published will not identify you personally. Your medical records will only be available to the research doctors, your hospital consultant, responsible individuals from the Cancer Research UK Clinical Trials Unit (Glasgow), trial sponsors and the regulatory authority.

With your permission, we will also inform your general practitioner of your participation in this trial.

For peer review only - http://bmjopen.bmj.com/site/about/guidelines.xhtml

With your permission, the Cancer Research UK Clinical Trials Unit (Glasgow) who co-ordinate the participation in the trial, will collect your name or initials, date of birth and NHS number or Community Health Index (CHI) number. This information will be stored securely on a password-protected database and will be kept strictly confidential, with access provided only to authorised personnel. This information will utilize NHS data for future research through Cancer Research UK, who are funding this trial.

Your consent for participation in this trial also includes your consent to allow the use of the data in your medical/clinical record to be used for the purposes of cancer research. Your consent also includes allowing this data to be linked to data coming from other sources such as cancer registries and medical clinical records. All data (personal, clinical, economic and data coming from research on biological material) collected on your behalf will be treated in compliance with the European and UK applicable laws to ensure your confidentiality is maintained.

What will happen to the results of the research trial?
The results of this trial will be presented at medical meetings and submitted to major cancer research journals for publication. You will not be identified in any way in any report or publication arising from the trial. Results will also be available through http://cancerbackup.org.uk

The results of this trial will also be presented outside the European Union. These areas may have fewer rules about data protection. However, you would never be identified individually during these presentations. Data sent to other groups in the UK and abroad will not include information that identifies you by name, and agreements will ensure the data is treated confidentially.

Who is organising and funding the research?
The research is being sponsored by NHS Greater Glasgow and Clyde and is funded by Cancer Research UK. The trial is being co-ordinated by the Cancer Research UK Clinical Trials Unit, Glasgow on behalf of the NHS Greater Glasgow and Clyde. The Chief Investigator is Dr Matthew Hatton, Consultant Clinical Oncologist at Weston Park Hospital, Sheffield.

Will I be paid any costs and reimbursements?
There will be no payment to you for entering this study, no reimbursement of expenses and no payment for undergoing investigations that are additional to your standard care.

Who has reviewed this trial?
This clinical trial has been reviewed by number of medical specialists during its development; the National Cancer Research Institute Lung Clinical Studies Group (NCRI Lung CSG) and the Clinical Trials Advisory and Awards Committee in the UK (CTAAC).

For peer review only - http://bmjopen.bmj.com/site/about/guidelines.xhtml

All research in the NHS is looked at by an independent group of people, called a Research Ethics Committee, to protect your interests. This trial has been reviewed and given a favourable opinion by the West of Scotland Research Ethics Committee.

Further information and contact details

If you consent to join this trial you will be given the telephone number of the hospital, which you can contact at any time if you feel unwell or have questions. We would like you to keep this information sheet and if you find it helpful please use it to discuss treatment with your family, friends or your General Practitioner. If you have any further questions or concerns regarding the trial please contact:

Doctor:

**Name**	**Insert local details**
**Telephone Number**	**Insert local details** (During office hours) or if out of office hours call **(Insert Local Out of Hours/Emergency Contact Details)**

Research Nurse:

**Name**	**Insert local details**
**Telephone Number**	**Insert local details**

If you would like independent advice or further information you may also find it useful to contact;

Cancerhelp UK (Cancer Research UK), who provide a wide range of information for people with cancer: Freephone: 0808 800 4040, and website: www.cancerhelp.org.uk

Macmillan Cancer Support, an independent patient advisory group: Freephone 0808 808 00 00; website www.macmillan.org.uk, Head Office Address: Macmillan Cancer Support, 89 Albert Embankment, London, SE1 7UQ.

Thank you for taking the time to read this information sheet and consider this trial. If you wish to take part you will be given a copy of this information sheet and a signed consent form to keep.

For peer review only - http://bmjopen.bmj.com/site/about/guidelines.xhtml

ADSCaN PARTICIPANT CONSENT FORM

Patient ID Number for this trial: Date:

Name of Principal Investigator:

Please initial box

1. I confirm that I have read and understand the information sheet dated (Version XXX Date XXX) for the above trial and have had the opportunity to ask questions.

2. I understand that my participation is voluntary and that I am free to withdraw at any time, without giving any reason, without my medical care or legal rights being affected.

3. I agree that relevant sections of any of my medical notes and data collected during the trial may be looked at by responsible individuals from the Cancer Research UK Clinical Trials Unit (Glasgow), the trial sponsors, the NHS organisation (hospital site) and the regulatory authorities where it is relevant to my taking part in research. I give permission for these individuals to have access to my records.

4. I consent to the storage of personal information (including electronic) for the purposes of this trial. I understand that any information that could identify me will be kept strictly confidential and that no personal information will be included in the trial report or other publication.

5. I agree to give permission for data collected relating to me to be used for "cancer research" purposes as described in this information sheet including allowing this data to be linked to data coming from other sources such as cancer registries and medical clinical records. I understand giving consent to the use of this data as described is optional and not mandatory for participating in this trial.

6. I give permission for my initials, date of birth and NHS (or Community Health Index (CHI)) number to be collected by the Cancer Research UK Clinical Trials Unit (Glasgow), where they will be stored in a secure location (including electronic). I understand giving consent to the use of this data as described is optional and not mandatory for participating in this trial.

7. I give permission for my GP to be informed of my inclusion in this trial.

For peer review only - http://bmjopen.bmj.com/site/about/guidelines.xhtml

8. I agree to give extra samples of blood and tissue for translational research purposes as described in the information sheet for this trial. I understand how the samples will be collected, that giving samples is voluntary and that I am free to withdraw my approval for use of the samples at any time without giving a reason and without my medical care or legal rights being affected **(optional)**. ☐

9. I agree that the blood and tissue samples and information collected about me will be stored on behalf of the ADSCaN Trial Management Group and may be used in future projects, as described in the information sheet. I understand that some of these projects may be carried out by researchers other than the ADSCaN Trial Management Group **(optional)**. ☐

10. I understand that I shall not benefit financially if future research leads to the development of new treatments or medical tests. ☐

11. I agree to the information detailed in the information sheet to be collected as part of the trial. ☐

12. I agree to take part in the ADSCaN trial. ☐

Name of Patient	Date	Signature
Name of person taking consent	Date	Signature

1 for patient; 1 for researcher; 1 to be kept with hospital notes

For peer review only

For peer review only - http://bmjopen.bmj.com/site/about/guidelines.xhtml

STANDARD PROTOCOL ITEMS: RECOMMENDATIONS FOR INTERVENTIONAL TRIALS

SPIRIT 2013 Checklist: Recommended items to address in a clinical trial protocol and related documents*

Section/item	Item No	Description	Addressed on page number
**Administrative information**			
Title	1	Descriptive title identifying the study design, population, interventions, and, if applicable, trial acronym	1
Trial registration	2a	Trial identifier and registry name. If not yet registered, name of intended registry	2
	2b	All items from the World Health Organization Trial Registration Data Set	NA
Protocol version	3	Date and version identifier	9
Funding	4	Sources and types of financial, material, and other support	12
Roles and responsibilities	5a	Names, affiliations, and roles of protocol contributors	1, 12
	5b	Name and contact information for the trial sponsor	12
	5c	Role of study sponsor and funders, if any, in study design; collection, management, analysis, and interpretation of data; writing of the report; and the decision to submit the report for publication, including whether they will have ultimate authority over any of these activities	12
	5d	Composition, roles, and responsibilities of the coordinating centre, steering committee, endpoint adjudication committee, data management team, and other individuals or groups overseeing the trial, if applicable (see Item 21a for data monitoring committee)	10,12

For peer review only
For peer review only - http://bmjopen.bmj.com/site/about/guidelines.xhtml

**Introduction**			
Background and rationale	6a	Description of research question and justification for undertaking the trial, including summary of relevant studies (published and unpublished) examining benefits and harms for each intervention	3
	6b	Explanation for choice of comparators	3-5
Objectives	7	Specific objectives or hypotheses	5
Trial design	8	Description of trial design including type of trial (eg, parallel group, crossover, factorial, single group), allocation ratio, and framework (eg, superiority, equivalence, noninferiority, exploratory)	5
**Methods: Participants, interventions, and outcomes**			
Study setting	9	Description of study settings (eg, community clinic, academic hospital) and list of countries where data will be collected. Reference to where list of study sites can be obtained	6
Eligibility criteria	10	Inclusion and exclusion criteria for participants. If applicable, eligibility criteria for study centres and individuals who will perform the interventions (eg, surgeons, psychotherapists)	7
Interventions	11a	Interventions for each group with sufficient detail to allow replication, including how and when they will be administered	13
	11b	Criteria for discontinuing or modifying allocated interventions for a given trial participant (eg, drug dose change in response to harms, participant request, or improving/worsening disease)	7, 8
	11c	Strategies to improve adherence to intervention protocols, and any procedures for monitoring adherence (eg, drug tablet return, laboratory tests)	NA
	11d	Relevant concomitant care and interventions that are permitted or prohibited during the trial	NA
Outcomes	12	Primary, secondary, and other outcomes, including the specific measurement variable (eg, systolic blood pressure), analysis metric (eg, change from baseline, final value, time to event), method of aggregation (eg, median, proportion), and time point for each outcome. Explanation of the clinical relevance of chosen efficacy and harm outcomes is strongly recommended	5
Participant timeline	13	Time schedule of enrolment, interventions (including any run-ins and washouts), assessments, and visits for participants. A schematic diagram is highly recommended (see Figure)	8

For peer review only - http://bmjopen.bmj.com/site/about/guidelines.xhtml

Sample size	14	Estimated number of participants needed to achieve study objectives and how it was determined, including clinical and statistical assumptions supporting any sample size calculations	5, 9
Recruitment	15	Strategies for achieving adequate participant enrolment to reach target sample size	3

Methods: Assignment of interventions (for controlled trials)

Allocation:			
Sequence generation	16a	Method of generating the allocation sequence (eg, computer-generated random numbers), and list of any factors for stratification. To reduce predictability of a random sequence, details of any planned restriction (eg, blocking) should be provided in a separate document that is unavailable to those who enrol participants or assign interventions	7
Allocation concealment mechanism	16b	Mechanism of implementing the allocation sequence (eg, central telephone; sequentially numbered, opaque, sealed envelopes), describing any steps to conceal the sequence until interventions are assigned	7
Implementation	16c	Who will generate the allocation sequence, who will enrol participants, and who will assign participants to interventions	7
Blinding (masking)	17a	Who will be blinded after assignment to interventions (eg, trial participants, care providers, outcome assessors, data analysts), and how	NA
	17b	If blinded, circumstances under which unblinding is permissible, and procedure for revealing a participant's allocated intervention during the trial	NA

Methods: Data collection, management, and analysis

Data collection methods	18a	Plans for assessment and collection of outcome, baseline, and other trial data, including any related processes to promote data quality (eg, duplicate measurements, training of assessors) and a description of study instruments (eg, questionnaires, laboratory tests) along with their reliability and validity, if known. Reference to where data collection forms can be found, if not in the protocol	9
	18b	Plans to promote participant retention and complete follow-up, including list of any outcome data to be collected for participants who discontinue or deviate from intervention protocols	8

For peer review only - http://bmjopen.bmj.com/site/about/guidelines.xhtml

Data management	19	Plans for data entry, coding, security, and storage, including any related processes to promote data quality (eg, double data entry; range checks for data values). Reference to where details of data management procedures can be found, if not in the protocol	9
Statistical methods	20a	Statistical methods for analysing primary and secondary outcomes. Reference to where other details of the statistical analysis plan can be found, if not in the protocol	9
	20b	Methods for any additional analyses (eg, subgroup and adjusted analyses)	NA
	20c	Definition of analysis population relating to protocol non-adherence (eg, as randomised analysis), and any statistical methods to handle missing data (eg, multiple imputation)	NA
**Methods: Monitoring**			
Data monitoring	21a	Composition of data monitoring committee (DMC); summary of its role and reporting structure; statement of whether it is independent from the sponsor and competing interests; and reference to where further details about its charter can be found, if not in the protocol. Alternatively, an explanation of why a DMC is not needed	10
	21b	Description of any interim analyses and stopping guidelines, including who will have access to these interim results and make the final decision to terminate the trial	8
Harms	22	Plans for collecting, assessing, reporting, and managing solicited and spontaneously reported adverse events and other unintended effects of trial interventions or trial conduct	10
Auditing	23	Frequency and procedures for auditing trial conduct, if any, and whether the process will be independent from investigators and the sponsor	10
**Ethics and dissemination**			
Research ethics approval	24	Plans for seeking research ethics committee/institutional review board (REC/IRB) approval	2, 12
Protocol amendments	25	Plans for communicating important protocol modifications (eg, changes to eligibility criteria, outcomes, analyses) to relevant parties (eg, investigators, REC/IRBs, trial participants, trial registries, journals, regulators)	NA

For peer review only - http://bmjopen.bmj.com/site/about/guidelines.xhtml

Consent or assent	26a	Who will obtain informed consent or assent from potential trial participants or authorised surrogates, and how (see Item 32)	6
	26b	Additional consent provisions for collection and use of participant data and biological specimens in ancillary studies, if applicable	NA
Confidentiality	27	How personal information about potential and enrolled participants will be collected, shared, and maintained in order to protect confidentiality before, during, and after the trial	
Declaration of interests	28	Financial and other competing interests for principal investigators for the overall trial and each study site	10
Access to data	29	Statement of who will have access to the final trial dataset, and disclosure of contractual agreements that limit such access for investigators	9
Ancillary and post-trial care	30	Provisions, if any, for ancillary and post-trial care, and for compensation to those who suffer harm from trial participation	NA
Dissemination policy	31a	Plans for investigators and sponsor to communicate trial results to participants, healthcare professionals, the public, and other relevant groups (eg, via publication, reporting in results databases, or other data sharing arrangements), including any publication restrictions	9
	31b	Authorship eligibility guidelines and any intended use of professional writers	NA
	31c	Plans, if any, for granting public access to the full protocol, participant-level dataset, and statistical code	11
**Appendices**			
Informed consent materials	32	Model consent form and other related documentation given to participants and authorised surrogates	Appendix
Biological specimens	33	Plans for collection, laboratory evaluation, and storage of biological specimens for genetic or molecular analysis in the current trial and for future use in ancillary studies, if applicable	NA

*It is strongly recommended that this checklist be read in conjunction with the SPIRIT 2013 Explanation & Elaboration for important clarification on the items. Amendments to the protocol should be tracked and dated. The SPIRIT checklist is copyrighted by the SPIRIT Group under the Creative Commons "Attribution-NonCommercial-NoDerivs 3.0 Unported" license.

For peer review only - http://bmjopen.bmj.com/site/about/guidelines.xhtml
